# Novel mutation in hexokinase 2 confers resistance to 2-deoxyglucose by altering protein dynamics

**DOI:** 10.1371/journal.pcbi.1009929

**Published:** 2022-03-02

**Authors:** Erich Hellemann, Jennifer L. Walker, Mitchell A. Lesko, Dakshayini G. Chandrashekarappa, Martin C. Schmidt, Allyson F. O’Donnell, Jacob D. Durrant

**Affiliations:** 1 Department of Biological Sciences, University of Pittsburgh, Pittsburgh, Pennsylvania, United States of America; 2 University of Pittsburgh School of Medicine, Department of Microbiology and Molecular Genetics, University of Pittsburgh, Pittsburgh, Pennsylvania, United States of America; US Army Medical Research and Materiel Command: US Army Medical Research and Development Command, UNITED STATES

## Abstract

Glucose is central to many biological processes, serving as an energy source and a building block for biosynthesis. After glucose enters the cell, hexokinases convert it to glucose-6-phosphate (Glc-6P) for use in anaerobic fermentation, aerobic oxidative phosphorylation, and the pentose-phosphate pathway. We here describe a genetic screen in *Saccharomyces cerevisiae* that generated a novel spontaneous mutation in hexokinase-2, *hxk2*^G238V^, that confers resistance to the toxic glucose analog 2-deoxyglucose (2DG). Wild-type hexokinases convert 2DG to 2-deoxyglucose-6-phosphate (2DG-6P), but 2DG-6P cannot support downstream glycolysis, resulting in a cellular starvation-like response. Curiously, though the *hxk2*^G238V^ mutation encodes a loss-of-function allele, the affected amino acid does not interact directly with bound glucose, 2DG, or ATP. Molecular dynamics simulations suggest that Hxk2^G238V^ impedes sugar binding by altering the protein dynamics of the glucose-binding cleft, as well as the large-scale domain-closure motions required for catalysis. These findings shed new light on Hxk2 dynamics and highlight how allosteric changes can influence catalysis, providing new structural insights into this critical regulator of carbohydrate metabolism. Given that hexokinases are upregulated in some cancers and that 2DG and its derivatives have been studied in anti-cancer trials, the present work also provides insights that may apply to cancer biology and drug resistance.

## Author summary

Glucose fuels many of the energy-production processes required for normal cell growth. Before glucose can participate in these processes, it must first be chemically modified by proteins called hexokinases. To better understand how hexokinases modify glucose—and how mutations in hexokinase genes might confer drug resistance—we evolved resistance in yeast to a toxic hexokinase-binding molecule called 2DG. We discovered a mutation in the hexokinase gene that confers 2DG resistance and reduces the protein’s ability to modify glucose. Biochemical analyses and computer simulations of the hexokinase protein suggest that the mutation diminishes glucose binding by altering enzyme flexibility. This work shows how cells can evolve resistance to toxins via only modest changes to protein structures. Furthermore, because cancer-cell hexokinases are particularly active, 2DG has been studied as cancer chemotherapy. Thus, the insights this work provides might also apply to cancer biology.

## Introduction

Eukaryotic cells sense and respond to their nutrient environment, modulating their metabolic processes to grow and survive in a changing nutritional landscape. The available carbohydrate source dictates these metabolic shifts. Glucose, a six-carbon sugar critical for many biological processes, is the preferred carbon source for most eukaryotic cells. Glucose is first taken up from the environment by glucose transporters (e.g., the HXT hexose transporters in *S*. *cerevisiae* or the homologous GLUT glucose transporters in mammals). Catabolism then begins with the activity of a hexokinase, which transfers the γ phosphate of ATP to the C6 carbon of glucose, producing glucose-6-phosphate (Glc-6P) [1]. Through a series of enzymatic conversions, glycolysis ultimately converts Glc-6P into two pyruvate molecules, which are further catabolized via anaerobic fermentation and aerobic oxidative phosphorylation (OXPHOS) to produce the energy needed for cellular function [2]. Glucose-dependent reductive biosynthesis occurs in parallel, primarily through the pentose-phosphate pathway. The same hexokinase-generated Glc-6P molecules serve as initial building blocks to generate compounds such as NADPH and ribose 5-phosphate, which are in turn the precursors for many biosynthetic processes (e.g., nucleic-acid and fatty-acid synthesis) [3–5]. Because Glc-6P feeds into several glucose pathways, hexokinase enzymes are critical regulatory checkpoints [4, 6].

**Fig 1:**
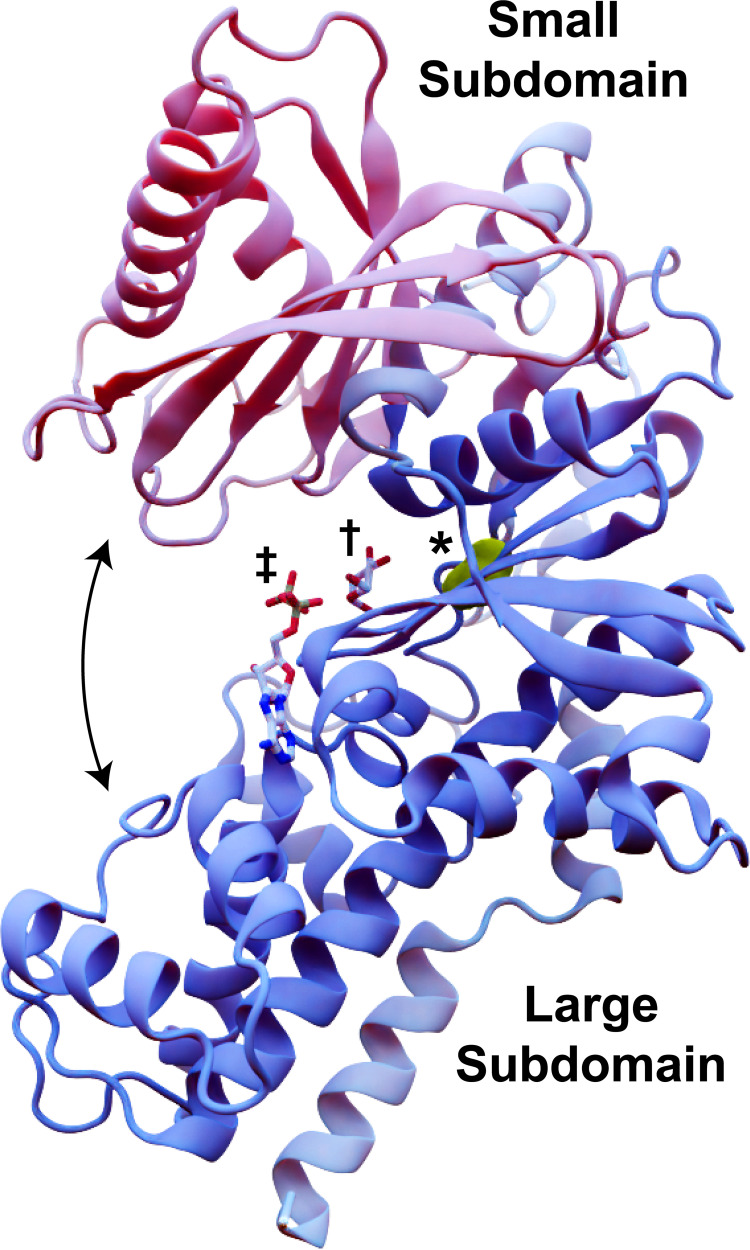
*Sc*Hxk2 structure and global dynamics. The mostly helical large subdomain and the ɑ/β small subdomain are shown in blue and pink, respectively (PDB 1IG8). The curved arrows indicate the approximate motion of domain closure. The location of residue 238 is shown in yellow and marked with an asterisk. To indicate the location of the glucose- and ATP-binding pockets, we superimposed crystallographic glucose (dagger) and ADP (double dagger) molecules from structures of *Hs*Hk1 (human, PDB 4FPB) and *Os*Hxk6 (rice, PDB 6JJ8), respectively.

The conformational changes that hexokinases undergo as they engage with substrate have been extensively studied [7–14]. Hexokinases generally adopt a palm-shaped ɑ/β fold with at least two subdomains: a mostly helical large subdomain and an ɑ/β small subdomain (Fig 1). The enzyme starts in an open conformation, such that the central inter-domain crevice (i.e., the enzymatic cleft) is accessible to bulk solvent and substrate [7, 8]. Glucose binding to the cleft causes the two subdomains to rotate relative to each other [9, 10]. This rotation collapses the solvent-accessible crevice, leading it to envelop the glucose molecule [7, 11] via a so-called “embracing” mechanism [12]. ATP binding induces further conformational changes [11, 13] that allow an amino acid acting as a catalytic base (D211 in yeast Hxk2) to abstract a hydrogen atom from the glucose 6-oxygen, enabling nucleophilic attack on the ATP γ phosphorus [14]. Electrostatic interactions between the resulting Glc-6P and ADP drive the two products apart, leading to the release of Glc-6P.

While the mechanics of glucose binding and catalysis have been extensively studied in several species (e.g., *H*. *sapiens* [15], *K*. *lactis* [16], and *S*. *cerevisiae* [7]), it remains to be determined how glucose analogs impact those mechanisms. We focus on 2-deoxyglucose (2DG), a toxic glucose analog studied as an anti-cancer agent in clinical trials. 2DG is an excellent chemical probe for studying glucose metabolism, hexokinase enzymology, and resistance mechanisms. It is identical to glucose, except a hydrogen atom is present at the C2 carbon rather than a hydroxyl group. Because of these chemical similarities, studying the impact of 2DG on cellular function can reveal insights into wild-type (WT) glucose metabolic mechanisms.

After 2DG enters the cell via glucose transporters, it too is phosphorylated by hexokinases, producing 2-deoxyglucose-6-phosphate (2DG-6P) [17]. Unlike Glc-6P, 2DG-6P cannot advance through the glycolytic pathway [18, 19] and even inhibits some glycolytic enzymes (e.g., hexokinase [20] and glucose-6-phosphate isomerase [21]). Aside from blocking glycolysis, 2DG also acts via several other toxic mechanisms. For example, it may deplete cellular ATP reserves because 2DG phosphorylation consumes ATP, but 2DG-6P cannot be recycled or used for energy production [22]. 2DG also incorporates into glycolipids, endogenous molecules that play critical roles in many cellular pathways [23]. Resulting downstream metabolites (e.g., GDP-2DG and UDP-2DG) also impact glycogen metabolism [24] and protein glycosylation [25]. The latter results in protein misfolding and endoplasmic reticulum stress, which can trigger the unfolded protein response and apoptosis [26, 27]. In yeast, 2DG also compromises cell-wall integrity; altered protein glycosylation impacts mannan biosynthesis [28], and 2DG incorporates into cell-wall β-glucan polymers [29]. Additional mechanisms of toxicity are also likely [30–33], and much remains uncertain [18, 34]. See Laussel et al. [35] for a thorough review.

Yeast is an excellent organism for studying 2DG biology and resistance mechanisms. As early as the 1960s, researchers noted that when S. cerevisiae is grown in media containing 0.2% 2DG, it exhibits a glucose-starvation phenotype even when 2% glucose is present [36, 37]. Yeast cells exposed to 2DG also acquire resistance, and the organism’s well-characterized, small, and optionally haploid genome enables the rapid evolution and identification of resistance-conferring mutations [38]. For example, two recent genetic screens [39, 40] identified several critical contributors to 2DG resistance, including glucose-transporter trafficking, altered signaling through the AMP-activated kinase (AMPK in mammals, Snf1 in yeast), and changes in the unfolded protein response or cell integrity pathways. Mutations that dampen the catalytic activity of hexokinase-2 (*Sc*Hxk2), the predominant glucose kinase in high-glucose conditions [41], can also confer 2DG resistance [40]. Indeed, Hxk2 is the only one of three hexokinase isozymes in yeast (Hxk1, Hxk2, and Glk1) implicated in 2DG resistance, even though Hxk1 (and possibly Glk1) can also phosphorylate 2DG [40]. However, the data to date do not provide any atomic-resolution models for how Hxk2 mutations promote 2DG resistance.

In the present work, we use *in vivo* evolution and whole genome sequence analysis as an unbiased approach to identify spontaneous 2DG-resistance mutations in S. cerevisiae. We identify a novel resistance-conferring mutation in the hexokinase-2 gene (*HXK2*). Curiously, the mutation alters an amino acid that does not immediately line the enzymatic cleft, nor does it disrupt the stability of the enzyme. We use molecular, biochemical, and genetic experiments coupled with atomic-resolution molecular dynamics (MD) simulations to provide evidence that this novel mutation diminishes Hxk2 catalytic activity by indirectly impacting (1) local cleft dynamics and (2) the larger conformational changes required to envelop hexose substrates (e.g., glucose, 2DG). Our study illustrates how intra-protein allosteric communication can substantially impact overall activity and dynamics. Further, our findings provide generalizable insights into hexokinase resistance mechanisms that may be relevant to cancer biology.

## Results

### Directed evolution evolves resistance to 2DG

To identify novel resistance mechanisms, we used the uniquely sensitive ABC16-monster S. cerevisiae strain (also known as ΔABC16), which lacks 16 distinct ABC transporters [38, 42–45]. Because cells from this background are less able to evade cytotoxic chemicals by simple export, resistance is more likely to occur through compensatory mutations, often in the very protein(s) to which a cytotoxic compound binds. In five independent lab-evolution experiments, we exposed ABC16-monster cells to escalating 2DG concentrations via serial passaging to select for cells that evolved 2DG resistance (referred to hereafter as 2DG-resistant strains 1–5; see Materials and Methods for details).

**Fig 2:**
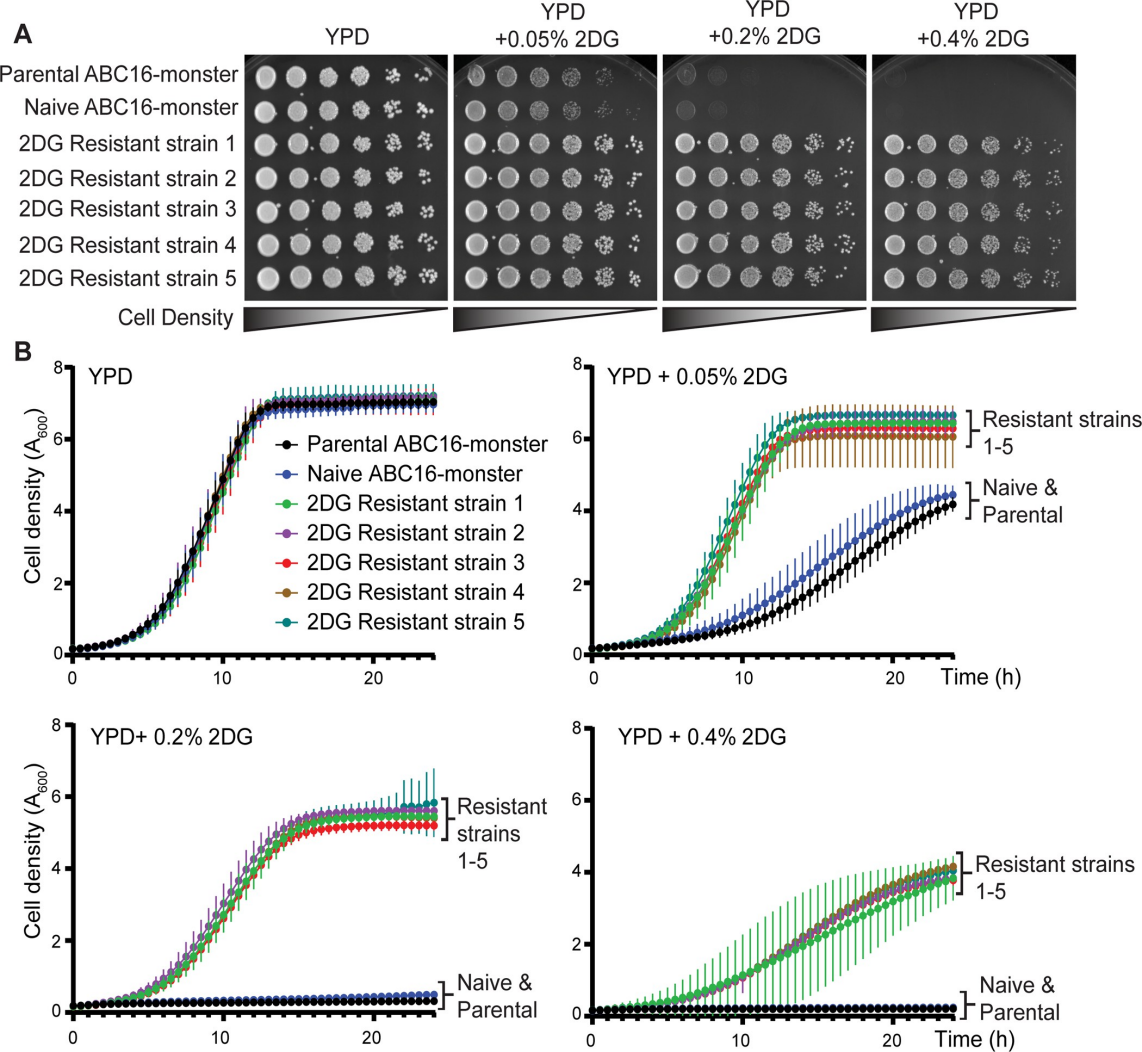
Cells from 2DG-resistant strains 1–5 are resistant to 2DG, but the parental control and naïve control cells are not. (A) Images of serial dilution growth assays on YPD (2% glucose as carbon source) or YPD with increasing concentrations of 2DG (0.05%, 0.2%, and 0.4%) after two days of growth at 30°C. (B) Graphs showing the change in cell density over time for cells grown in YPD or YPD with increasing 2DG concentrations, each of which have 2% glucose as a carbon source. Data are plotted as the A_600_ measured every 30 minutes during a 24-hour time course. Curves represent the average A_600_ of three experimental replicates, and vertical lines emerging from each data point represent standard deviations.

To verify 2DG resistance, we compared the growth of cells from the 2DG-resistant strains and two control strains: the parental ABC16-monster strain and an ABC16-monster strain that was passaged in parallel but without 2DG (referred to hereafter as naïve ABC16-monster; Fig 2). All strains grew robustly on YPD medium lacking 2DG, indicating that none of the mutations in the 2DG-resistant strains conferred a growth disadvantage in glucose (Fig 2A). In medium with 2DG, cells from the parental or naïve ABC16-monster strains grew poorly or failed to grow, depending on the 2DG concentration (Fig 2A). In contrast, cells from 2DG-resistant strains 1–5 grew at all 2DG concentrations tested (Fig 2A). Growth curve analyses of cells from the control strains (parental and naïve ABC-monster) and 2DG-resistant strains over 24 hours in liquid media showed similar trends (Fig 2B).

These assays reveal that the 2DG-resistant strains exhibit similar growth kinetics to the parental and naïve ABC16-monster controls on YPD (Fig 2B and Table 1). However, in the presence of 2DG, 2DG-resistant strains 1–5 had much shorter doubling times than the control strains (Fig 2B and Table 1). At the higher concentrations of 2DG (0.2% or 0.4%), it was impossible to calculate doubling times for the parental or naïve ABC16-monster strains because the cells did not grow at all (Fig 2B and Table 1). In contrast, for the 2DG resistant strains the doubling times, while longer than at lower 2DG concentrations, were possible to assess. Strikingly, the 2DG growth profiles for 2DG-resistant strains 1–5 were similar across all concentrations of 2DG tested (Fig 2B and Table 1). These findings confirm that our lab-evolution protocol successfully generated 2DG-resistant yeast cells.

**Table 1:** Doubling times calculated from the growth curves shown in Fig 2B (hours).

	Parental ABC16-monster	Naïve ABC16-monster	2DG-resistant strain 1	2DG-resistant strain 2	2DG-resistant strain 3	2DG-resistant strain 4	2DG-resistant strain 5
**YPD**	2.58 ± 0.19 (ns)	2.48 ± 0.31 (ns)	2.35 ± 0.14	2.60 ± 0.32 (ns)	2.47 ± 0.29 (ns)	2.39 ± 0.22 (ns)	2.51 ± 0.20 (ns)
**YPD + 0.05% 2DG**	5.34 ± 0.43 (***)	5.61 ± 0.20 (***)	2.85 ± 0.22	2.86 ± 0.03 (ns)	2.75 ± 0.06 (ns)	2.65 ± 0.09 (ns)	2.84 ± 0.07 (ns)
**YPD + 0.2% 2DG**	ND	ND	3.18 ± 0.21	3.28 ± 0.05 (ns)	3.20 ± 0.06 (ns)	3.19 ± 0.1 (ns)	3.25 ± 0.09 (ns)
**YPD + 0.4% 2DG**	ND	ND	5.09 ± 0.61	4.6 ± 0.42 (ns)	4.59 ± 0.16 (ns)	5.2 ± 0.33 (ns)	4.91 ± 0.27 (ns)

Doubling times are presented in hours for the average of three replicate experiments, plus or minus the standard deviation. Statistical analyses using ANOVA to compare the doubling times of each sample in a specific condition to the doubling time of 2DG-resistant strain 1 are also provided in parenthesis, where a *p*-value of ns = not significant and

*** < 0.0001. None of the 2DG resistant strains are significantly different from one another under any growth condition. The parental and naïve ABC-monster strains are also not statistically different from one another or the 2DG resistant strains in YPD. However, the parental and naïve ABC-monster strains are statistically different from the 2DG resistant strains when grown in even the lowest 2DG concentration (0.05%).

### A novel 2DG-resistance mutation in *HXK2*

To determine the genetic cause of this resistance, we used whole genome sequencing to identify any altered protein-encoding genes in 2DG-resistant strains 1–5 and to assess the ploidy of these strains (Fig A in S1 Text). Interestingly, all five evolved strains contained a missense mutation that substitutes valine for the glycine at Hxk2 amino acid 238 (i.e., Hxk2^G238V^) and bore no changes in ploidy, which can also be associated with 2DG resistance [46]. Although other hypomorphic *HXK2* alleles have been associated with 2DG resistance [18, 33, 39, 40], none have been identified in the ABC16-monster variant.

**Fig 3:**
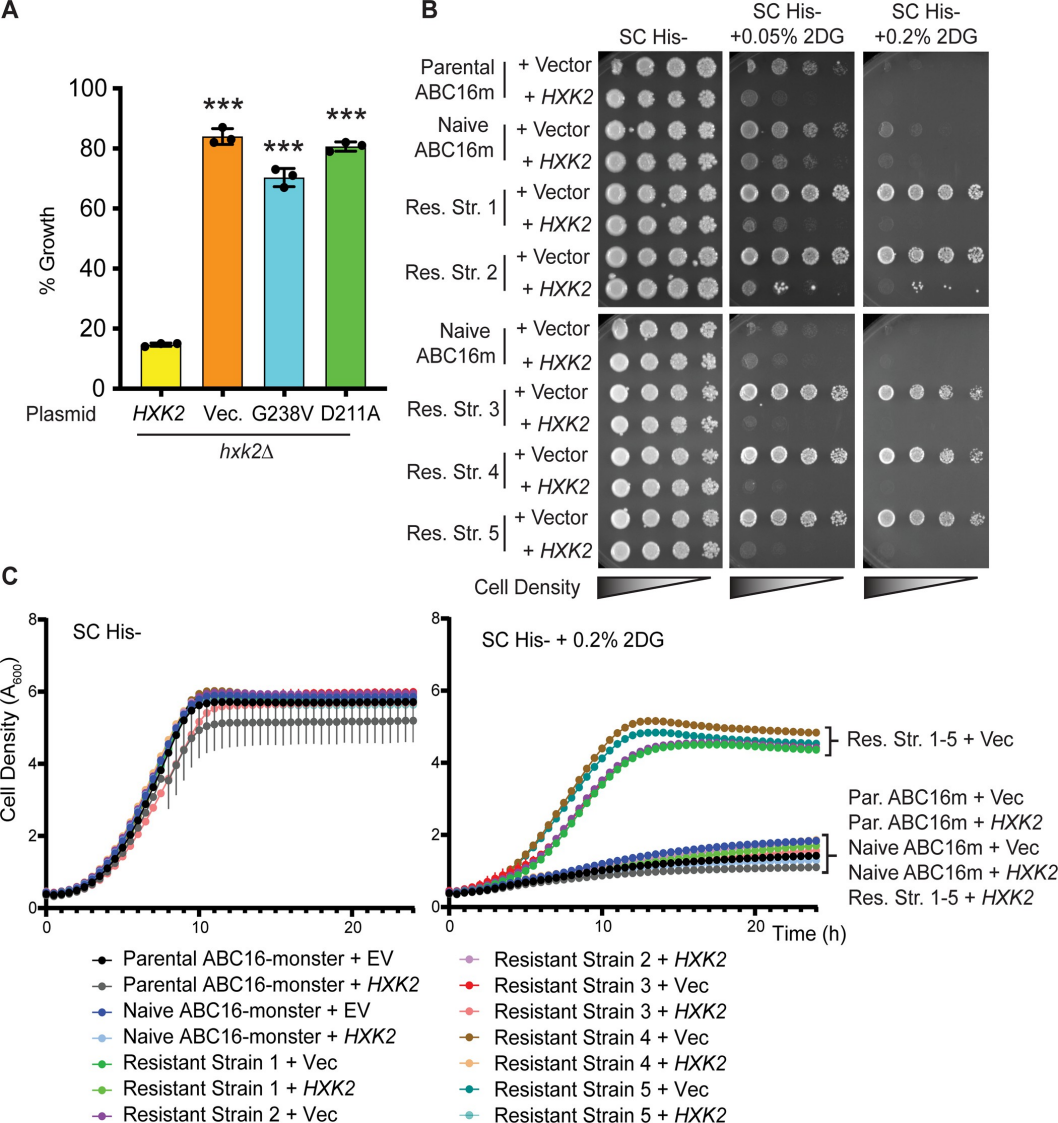
The *hxk2*^G238V^ mutation is sufficient to cause 2DG resistance. (A) 2DG-resistance assay of *hxk2*Δ cells with CEN plasmids expressing either WT *HXK2*, *hxk2*^G238V^, *hxk2*^D211A^, or pRS313 vector. Three independent cultures were grown in medium with 2% glucose and either with or without 0.1% 2DG. Percent growth for each replicate is plotted relative to growth in the absence of 2DG, and the error bars represent +/- SD. A Student’s t-test was used to compare *hxk2*Δ with the *HXK2*-expressing plasmids (*** indicates a *p*-value < 0.001). (B) Images of serial dilution growth plates containing cells from the parental or naïve ABC16-monster strain as well as 2DG-resistant strains 1–5, each transformed with pRS313 empty vector or a plasmid expressing WT *HXK2*, after two days of growth at 30°C. All media contains 2% glucose as carbon source and the indicated added 2DG. (C) Graphs showing the change in cell density over time for cells grown in SC-His- or SC-His- with increasing 2DG concentrations (as described in B). Data are plotted as the A_600_ measured every 30 minutes, correcting for a 1 cm path length. Curves represent the average A_600_ of three technical replicates, and vertical lines from each data point represent +/-SD. In many cases, the SD was so small it did not expand beyond the data point itself.

To confirm that the *hxk2*^G238V^ allele confers 2DG resistance, we introduced *hxk2*^G238V^ into cells lacking *HXK*2 (hxk2Δ), which are themselves resistant to 2DG. The cells employed in these confirmatory assays had the ABC transporters intact (i.e., they were not isogenic to the ABC16-monster). As expected, the presence of a WT *HXK2* restored sensitivity to 2DG (negative control, Fig 3A). However, adding vector, *hxk2*^G238V^, or the catalytically dead *hxk2*^D211A^ allele to *hxk2*Δ cells did not disrupt the 2DG resistance (Fig 3A).

While the *hxk2*^G238V^ allele is sufficient to cause 2DG resistance, each of the 2DG-resistant strains had other mutations that might also contribute to resistance [47]. To determine the extent of these contributions, we transformed WT *HXK2* on a plasmid into 2DG-resistant strains 1–5 and spotted the cells as serial dilutions. We found that 2DG sensitivity was completely restored in all five cases (Fig 3B), and growth kinetics in the presence of 2DG were comparable to the parental ABC16-monster strain (Fig 3C). This experiment suggests that the *hxk2*^G238V^ allele is primarily responsible for the observed 2DG resistance.

**Fig 4:**
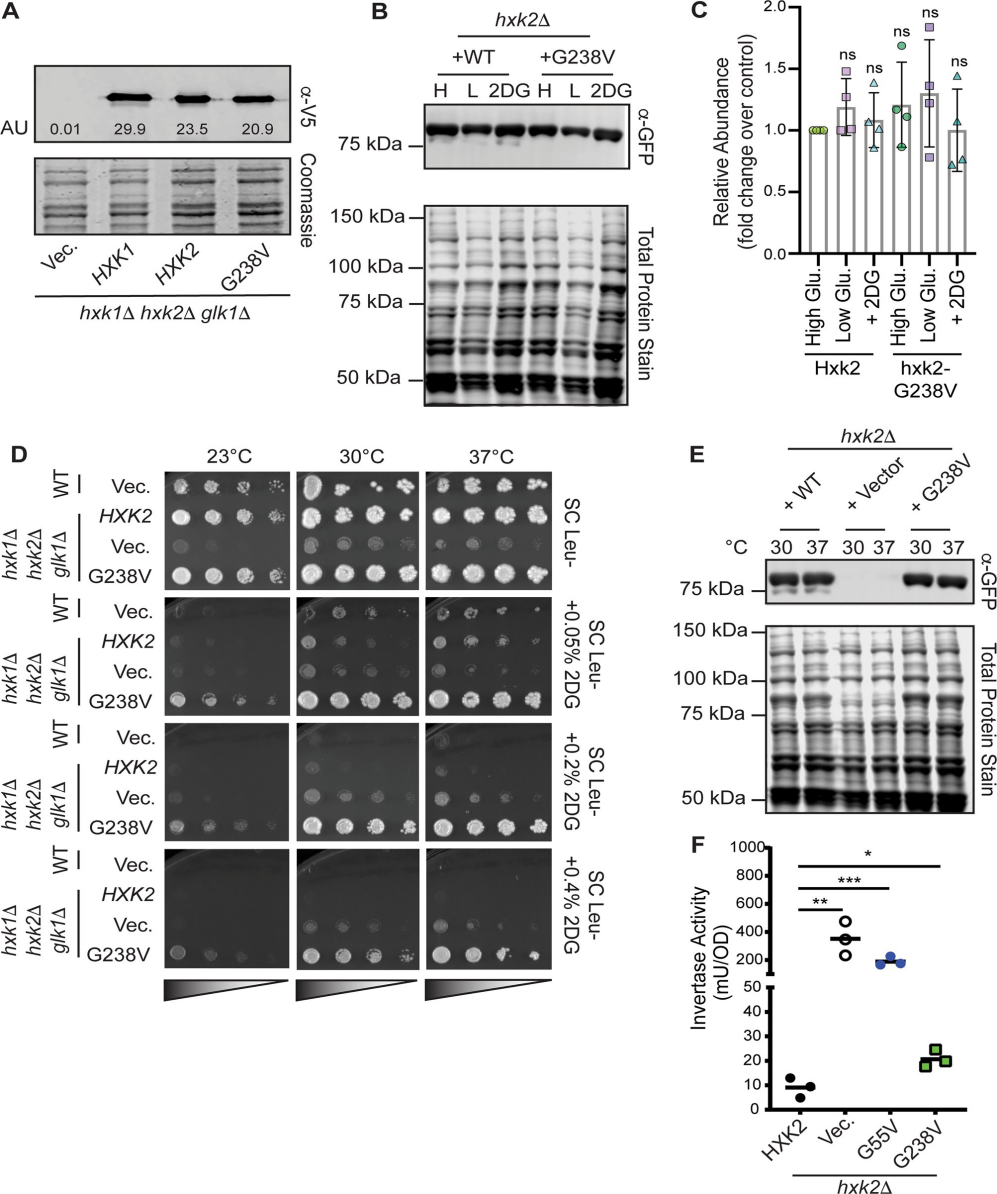
Hxk2^G238V^ is a stable protein that allows for growth on glucose in cells that otherwise lack hexokinase activity. (A) Western blot of V5-epitope-tagged *HXK1*, *HXK2*, and *hxk2*^G238V^ expressed in *hxk1*Δ *hxk2*Δ *glk1*Δ cells. Quantification of the Hxk2 western signal is depicted in arbitrary units (AU) on the blot. (B) Western blot showing GFP-tagged Hxk2 protein levels from whole-cell extracts prepared from *hxk2*Δ cells grown in synthetic complete media with 2% glucose (H for high glucose) or 0.05% glucose (L for low glucose) alone, or with 2% glucose and the addition of 0.2% 2DG for two hours (2DG for medium containing this drug). REVERT total protein stain was used as a protein loading control. (C) Quantification of western signal as shown in panel B for four experimental replicates. The abundance of Hxk2 in high glucose conditions was normalized to 1 in each experiment, and all other values are shown as a relative fold change. A Student’s t-test was performed to determine if any samples were different from the Hxk2 abundance in high glucose; none differed significantly from this standard. (D) Serial dilution spot assays of *hxk1*Δ *hxk2*Δ *glk1*Δ transformed with an empty vector control plasmid or one expressing Hxk2 or Hxk2^G238V^. Cells were grown on the indicated medium, each of which contained 2% glucose as a carbon source and either no 2DG or 0.05–0.4% 2DG. The results pictured show cells after two days of growth at 23°C, 30°C, or 37°C. (E) Western blot showing GFP-tagged Hxk2 protein abundance in whole-cell extracts prepared from *hxk2*Δ cells expressing WT Hxk2 and Hxk2^G238V^, from the same plasmids used in panel D. Cells were grown to mid-log phase in media containing 2% glucose before exposure to the indicated temperature for two hours. Proteins were then extracted. REVERT total protein stain was used as a protein loading control. (F) Invertase activity measured in three independent transformants of *hxk2*Δ cells grown in media with 2% glucose. A Student’s t-test was used to assess the statistical difference between experimental groups and the Hxk2-expressing control.

Because cells lacking *HXK2* entirely (hxk2Δ) are also resistant to 2DG, a trivial explanation for resistance is that the *hxk2*^G238V^ allele encodes an unstable protein product. We found that the steady-state protein abundance of Hxk2^G238V^ was comparable to that of two controls: WT Hxk2 and WT Hxk1 (Fig 4A). There was no statistically significant difference in Hxk2 or Hxk2^G238V^ abundance when cells were grown in high or low glucose conditions or in the presence of 2DG (Fig 4B and 4C), suggesting that the Hxk2^G238V^ variant is stable under all these conditions.

As further evidence of stability, we found that the *hxk2*^G238V^ allele supports growth on glucose in cells lacking all three hexokinases (*hxk2*Δ *hxk1*Δ *glk1*Δ), demonstrating that it is sufficiently folded and functional to convert enough glucose to Glc-6P to sustain life (Fig 4D, top panel). Indeed, even at elevated temperatures that unfold/destabilize metastable proteins encoded by some missense alleles [48, 49], Hxk2^G238V^ permitted growth on glucose while the vector control did not (Fig 4D). At high temperatures, Hxk2^G238V^ also continued to confer 2DG resistance and was as stable as WT Hxk2 (Fig 4D and 4E). These results show that the *hxk2*^*G238V*^ allele alone is sufficient to confer 2DG resistance and that it encodes a stable, functional hexokinase.

In addition to its enzymatic role, Hxk2 regulates gene expression as part of the glucose repression system. Specifically, Hxk2 is required for glucose repression of the *SUC2 gene*, which encodes the sucrose hydrolyzing enzyme invertase. Invertase activity thus serves as a valuable proxy for Hxk2-mediated regulatory activity [41]. Not all *HXK2* 2DG-resistance alleles alter Hxk2-mediated repression of *SUC2* expression and activity; while some are associated with aberrant *SUC2* expression, others have WT-like repression of invertase function [40]. In our assays, we found that cells with WT *HXK2* (control) fully repressed invertase activity; in contrast, cells with the *hxk2*^G238V^ allele were unable to fully repress activity, though they repressed it better than cells with (1) vector alone or (2) the 2DG-resistance *hxk2*^G55V^ allele (Fig 4F).

### Hxk2^G238V^ dampens glucose catalytic activity

**Fig 5:**
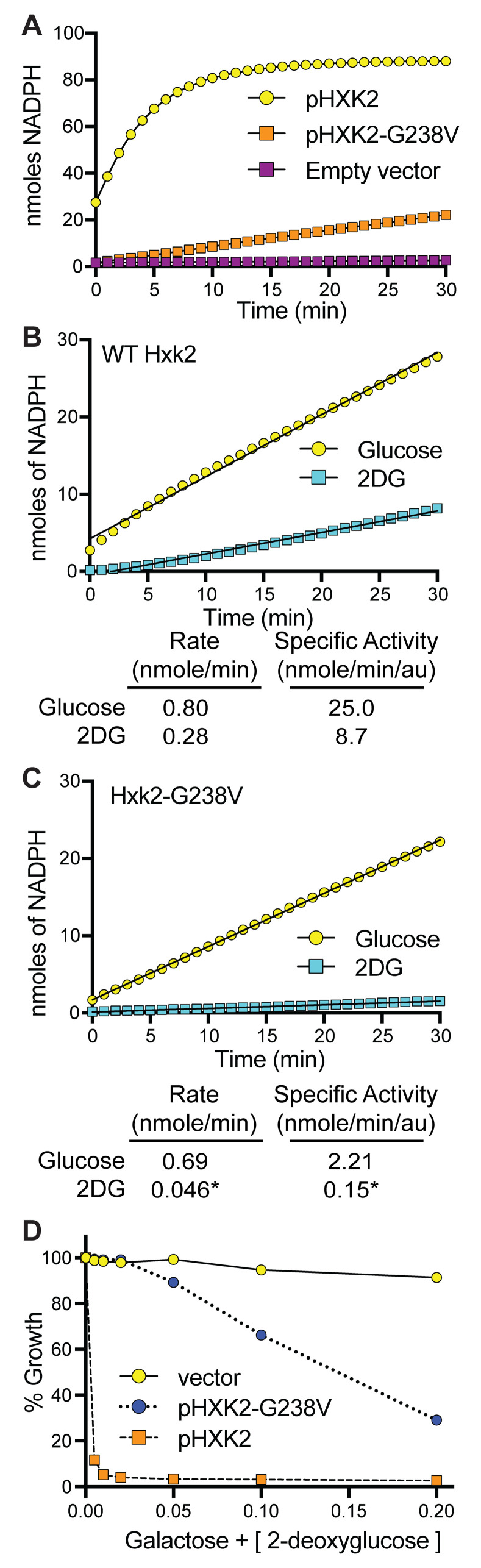
Hxk2^G238V^ has diminished enzymatic activity against glucose and 2DG. (A) Measure of NADPH production (a proxy for hexose phosphorylation) over time using 5 μg of yeast total protein extracts made from *hxk1*Δ *hxk2*Δ *glk1*Δ cells containing the plasmids indicated. Use of the empty vector serves as a negative control and demonstrates the specificity of this approach for the activity of the plasmid-borne Hxk2. (B-C) Rate of NADPH production in cells expressing WT Hxk2 (B) or Hxk2^G238V^ (C) when total protein extracts were incubated with glucose (yellow) or 2DG (blue). In panel B, 0.68 μg of yeast extract was used (0.032 au per reaction). In panel C, 5.2 μg of yeast extract was used (0.31 au per reaction) to allow the reduced activity of Hxk2^G238V^ to be detectable in our assays. Even with the elevated amount of enzyme used, the rate of NADPH production in Hxk2^G238V^ expressing extracts incubated with 2DG was too low to be accurately measured; the values provided are marked with * to indicate that they are not reliable measures above background. (D) Cells lacking endogenous hexokinase activity (*hxk1*Δ *hxk2*Δ *glk1*Δ) but containing an empty vector or plasmid expressing Hxk2 or Hxk2^G238V^ were grown in galactose and varying concentrations of 2DG. Hxk2^G238V^-expressing cells are more sensitive to 2DG than those lacking any Hxk2 activity at all, suggesting that Hxk2^G238V^ can phosphorylate 2DG *in vivo*.

To assess the impact of Hxk2^G238V^ on glucose phosphorylation, we assayed the enzymatic function of Hxk2^G238V^ and WT Hxk2 by adding a precisely defined concentration of glucose to yeast total protein extracts made from *hxk1*Δ *hxk2*Δ *glk1*Δ cells containing only plasmid-borne Hxk2^G238V^ or Hxk2. We then compared the average production of NADPH, which is a proxy for measuring glucose phosphorylation (see Materials and Methods). Such coupled enzyme assays are well established and validated; this particular hexokinase assay was first developed in the 1950’s [50] and has subsequently been cited over 450 times. These experiments revealed that Hxk2^G238V^ was a significantly less effective enzyme and had a lower affinity for glucose and ATP (Fig 5A and Table 2). The specific activity (V_max_/au) of WT Hxk2 for glucose is substantially higher than that of Hxk2^G238V^, while the K_m_ is substantially lower (Table 2, Fig BA and BB in S1 Text). The same trend holds for the K_m_ and specific activity of ATP (Table 2, Fig BC and BD in S1 Text). Yeast extract lacking all hexokinases (prepared using *hxk1*Δ *hxk2*Δ *glk1*Δ plus an empty vector) showed a very low background of Glc-6P conversion, serving as a negative control (Fig 5A). This low background confirms that other enzymes in the lysate that might similarly use ATP to reduce NADP to NADPH (e.g., galactokinase) are not active in our experiments due to the absence of their respective substrates (e.g., galactose).

**Table 2:** Enzyme kinetics for Hxk2 and Hxk2^G238V^.

	K_m_ Glucose (mM)	SA Glucose (nmole/min/au)	K_m_ 2DG (mM)	SA 2DG (nmole/min/au)	K_m_ ATP (mM)	SA ATP (nmole/min/au)
**WT Hxk2**	0.23 ± 0.02	27.6 ± 1.6	0.48 ± 0.06	8.59 ± 0.67	0.13 ± 0.01	24.2 ± 1.0
**Hxk2** ^**G238V**^	2.3 ± 0.37 (***)	8.9 ± 1.0 (**)	ND	ND	2.0 ± 0.21 (***)	7.1 ± 0.71 (**)

Each value is the mean of three experimental replicates ± the standard deviation. The raw data to support these measurements is presented in Fig B in S1 Text. Statistical analyses using a Student’s t-test to compare WT Hxk2 values to those of Hxk2^G238V^ are provided in parenthesis, where a *p*-value of ns = not significant

* < 0.05

** < 0.005, and

*** < 0.0005. The enzymatic activity of Hxk2^G238V^ against 2DG was not easily detectible above the background levels, so measurements were not determined (ND). However, Hxk2^G238V^ can clearly catalyze the conversion of 2DG because this allele elevates the sensitivity of galactose-grown cells to 2DG (see Fig 5D). K_m_ represents the Michaelis-Menten constant, and SA represents the specific activity (V_max_ normalized by the enzyme level).

Using a similar approach, we next examined the ability of Hxk2 and Hxk2^G238V^ to phosphorylate 2DG using protein extracts again made from yeast cells lacking all three hexokinases and expressing only Hxk2 or Hxk2^G238V^. We found that WT Hxk2 converted 2DG to 2DG-6P less effectively than it converted glucose to Glc-6P, as evidenced by a 2.5-fold reduction in the enzymatic rate and a 3-fold drop in specific activity with 2DG (Fig 5B and Table 2). Hxk2^G238V^ showed a similar trend, but Hxk2^G238V^ was less effective than WT Hxk2 at phosphorylating both glucose and 2DG (Fig 5C). The rate of Hxk2^G238V^ glucose phosphorylation was reduced to 0.69 nmole/min, consistent with what is reported in Table 2. Hxk2^G238V^ 2DG phosphorylation was so low that we were unable to reliably calculate the rate, specific activity, K_m_, or V_max_ (see notes in Fig 5B and Table 2).

To verify that Hxk2^G238V^ can still phosphorylate 2DG *in vivo*, we examined the physiological consequences of the allele in galactose-grown cells. We grew cells lacking the endogenous hexokinases (*hxk1*Δ *hxk2*Δ *glk1*Δ) and containing only plasmid-borne Hxk2 or Hxk2^G238V^ in galactose medium with increasing 2DG concentrations. We found that the cells with Hxk2^G238V^ were far more sensitive to 2DG than the vector control (Fig 5D). Because hexokinase activity is not needed for cell survival in galactose, this approach allowed us to selectively examine the impact of 2DG phosphorylation in the absence of glucose phosphorylation. These data support a model whereby Hxk2^G238V^ can phosphorylate both glucose and 2DG *in vivo*, but Hxk2^G238V^ is substantially less efficient than its WT Hxk2 counterpart.

### Summary of experimental results

In summary, the *hxk2*^G238V^ mutation, which we identified in all five evolved strains (2DG-resistant strains 1–5), is responsible for the observed 2DG resistance. We find that Hxk2^G238V^ is encoded by a stable yet hypomorphic allele of *HXK2*. Hxk2^G238V^ is as abundant as WT Hxk2 and provides the essential catalytic activity needed to allow hexokinase deletion cells to grow on glucose (i.e., it can convert glucose to Glc-6P), but its catalytic activity is nonetheless substantially lower than that of WT Hxk2. Importantly, unlike cells lacking all hexokinase activity (i.e., *hxk1*Δ *hxk2*Δ *glk1*Δ cells), Hxk2^G238V^-expressing cells are more sensitive to 2DG when grown in galactose, suggesting that Hxk2^G238V^ is still able to catalyze the conversion of 2DG to 2DG-6P *in vivo*.

### Hxk2^G238V^ is unlikely to interfere directly with substrate binding

To characterize the molecular underpinnings of *Sc*Hxk2^G238V^-mediated 2DG resistance and attenuated glucose phosphorylation, we first considered the possibility that the amino-acid change at position 238 directly interferes with substrate (e.g., 2DG or glucose) binding. A crystal structure of *Sc*Hxk2 in the open conformation (PDB 1IG8 [7]) reveals that G238 lies on a beta sheet (β10) near the glucose/2DG-binding site, but it does not line the site and so is unlikely to impede hexose association directly. We positioned a glucose molecule within the site by aligning a glucose-bound *Sc*Hxk1 structure (yeast, PDB 3B8A [8]) to 1IG8 [7]. The G238 residue is ~5.0 Å from the glucose substrate, and it participates in no apparent hydrogen-bond, electrostatic, or hydrophobic interactions with the sugar. The G238 side chain points away from the glucose molecule, so the larger V238 side chain in Hxk2^G238V^ is also unlikely to interact with the bound glucose.

To examine whether the amino acid at position 238 interacts with bound glucose when hexokinase is in the closed conformation—perhaps directly impeding the transfer of the γ phosphate from ATP—we examined a closed-state, glucose-bound structure of K. lactis Hxk1 (PDB 3O8M [16]). We chose this structure because there are no structures of glucose-bound *S*. cerevisiae Hxk2 in the Protein Data Bank [51, 52]. Fortunately, *Kl*Hxk1 is a good model for *Sc*Hxk2 [16] because the two proteins are highly homologous (73.4% amino-acid identity per Clustal Omega [53, 54], Fig C in S1 Text), have similar oligomerization [16], and are similarly phosphorylated at regulatory residue S15 [16]. In the closed-state 3O8M *Kl*Hxk1 structure, the bound glucose molecule is 5.8 Å from the G238-equivalent residue (also a glycine). Furthermore, G238 forms no interactions with the key catalytic residue D211, nor with residues known to interact with the bound glucose (S158, K176, E269, and E302) [7]. We therefore conclude that the G238V mutation is unlikely to impair phosphorylation by altering any of the direct interactions between *Sc*Hxk2^G238V^ and 2DG or glucose.

### *Sc*Hxk2^G238V^ may interfere with glucose binding by altering protein dynamics

To determine whether changes in protein dynamics might explain the attenuated enzymatic activity and 2DG resistance, we performed twelve molecular dynamics (MD) simulations of four *Sc*Hxk2 systems: WT Hxk2 apo (ligand-absent), Hxk2^G238V^ apo, WT Hxk2 holo (glucose-bound), and Hxk2^G238V^ holo. For each of the four systems, we performed three simulations of ~250 ns, ~250 ns, and ~500 ns each (~1 μS for each system, ~4 μS total). Unless otherwise noted, we treat the three simulations associated with each system as one. All simulations started from the same open conformation (PDB 1IG8 [7]); those that include bound glucose thus capture the initial dynamics associated with glucose association, including the large-scale conformational change (“domain closure,” Fig 1) required for catalysis.

### Hxk2^G238V^ impacts local pocket dynamics

**Fig 6:**
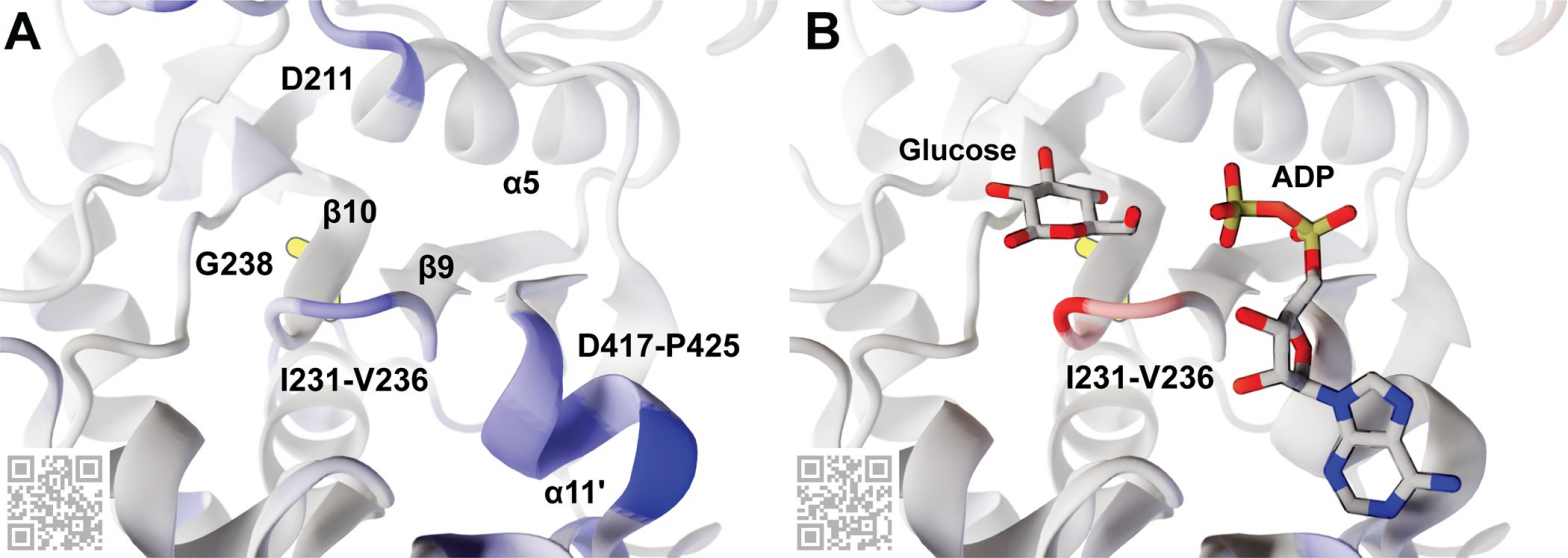
An Hxk2 pocket conformation taken from the MD simulations. G238, though largely obscured by the β10 strand, is shown in yellow sticks. Other key amino acids, molecules, and secondary-structure elements are labeled. The Hxk2 protein (ribbon) is colored according to the calculated ΔRMSF values, where blue indicates that the G238V simulation was more flexible (ΔRMSF > = 0.85 Å), and red indicates that the WT simulation was more flexible (ΔRMSF < = -0.85 Å). (A) The ΔRMSF values for the apo (ligand-absent) simulations. (B) The ΔRMSF values for the holo (glucose-bound) simulations. Glucose and ADP crystallographic poses are superimposed only for reference, to indicate the locations of the glucose and ATP-binding pockets. They were taken from structures of *Hs*HK1 (human, PDB 4FPB) and *Os*HXK6 (rice, PDB 6JJ8), respectively. QR codes encode ProteinVR URLs for visualization in stereoscopic 3D (virtual reality). See also Tables A and B in S1 Text.

**Fig 7:**
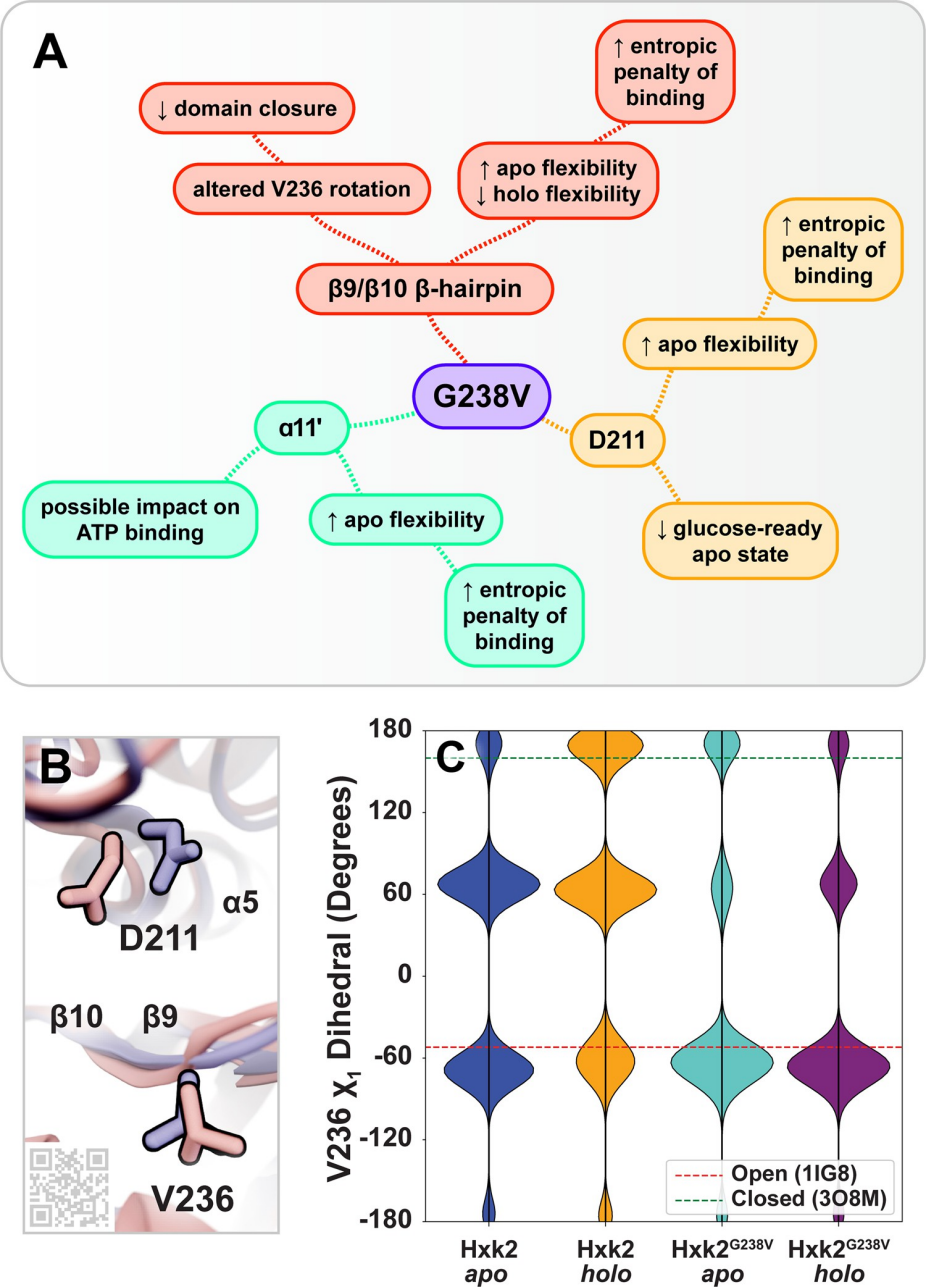
Hypothesized impacts of *Sc*Hxk2^G238V^ on the catalytic mechanism. (A) The G238V mutation alters the dynamics of the β9/β10 β-hairpin, the catalytic D211 residue, and the ɑ11’ helix. The implications of these changes on enzyme function are summarized. (B) Two conformations taken from the *Sc*Hxk2^G238V^ apo simulations highlight the movements of V236 (loop residue) and D211 (catalytic residue). In blue, V236 is shown in an open-like conformation, and D211 is shown in a glucose-ready conformation. In pink, V236 is shown in a closed-like conformation, and D211 is shown in a displaced conformation. Key secondary-structure elements are labeled with text, and the substrate-adjacent loop is labeled by its residues (I231-V236). The QR code provides a link to an online ProteinVR scene for virtual-reality visualization. (C) The distributions of the V236 χ_1_ dihedral angle. For reference, the red and green dashed lines show the corresponding values of the 1IG8 (*Sc*Hxk2, open) and 3O8M (*Kl*Hxk1, closed) crystal structures, respectively.

The simulations suggest that Hxk2^G238V^ influences three binding-cleft regions (Figs 6 and 7A): the β9/β10 β-hairpin (I231-V236), the catalytic residue D211, and the ɑ11’ helix (D417-P425). Specifically, Hxk2^G238V^ increases the apo-state flexibility of all three (Fig 6A, in blue ribbon) while stabilizing the holo-state β-hairpin (Fig 6B, in red ribbon). As detailed in the Discussion section, these changes in dynamics may impede glucose binding and phosphorylation, explaining the delayed Hxk2^G238V^ glucose catalysis we observed biochemically (Table 2 and Figs 5 and B in S1 Text).

#### β9/β10 β-hairpin (I231-V236)

Hxk2^G238V^ substantially impacts the dynamics of a β-hairpin loop that bridges the β9 and β10 strands (I231 to V236) [7]. This loop resides at the center of the enzymatic cleft near the glucose and ATP binding sites (Fig 6), so its dynamics likely influence the catalytic mechanism. To assess the flexibility of the β-hairpin loop residues, we calculated per-residue root-mean-square fluctuation (RMSF) and B-factor values (Tables S1 and A in S1 Text). To visualize the impact of the mutation, we mapped RMSF differences (ΔRMSF, RMSF_WT_—RMSF_G238V_) onto the protein structure (Fig 6). These ΔRMSF calculations suggest that in the *apo* (ligand-absent) state, the G238V mutation enhances the flexibility of the β-hairpin loop over WT; in contrast, in the *holo* (glucose-bound) state, the mutation reduces β-hairpin flexibility over WT (Tables S1 and A in S1 Text).

Hxk2^G238V^ also impacts the extent to which β-hairpin and residue-238 motions are correlated. We used dynamic cross correlation (DCC) to compare the motions of G238/V238 to those of the other Hxk2 residues. Tables S1 and A in S1 Text present per-residue differences in correlation coefficients (ΔDCC, DCC_WT_—DCC_G238V_). The motions of key β-hairpin residues and residue 238 are more correlated in Hxk2^G238V^ than in WT Hxk2 (Table A in S1 Text), suggesting an allosteric influence that is stronger in Hxk2^G238V^. Indeed, multiple β-hairpin residues have ΔDCC values more than two standard deviations from the mean ΔDCC across all residues (G235 and V236 in the apo simulations, and T234 and G235 in the *holo* simulations; Table A in S1 Text).

Hxk2^G238V^ also impacts the dynamics of β-hairpin-residue V236, which may influence the domain closure required for catalysis (see Discussion). We monitored the V236 χ_1_ dihedral angle (CG2-CB-CA-C) throughout the simulations. In the WT Hxk2 *apo* simulation, V236 heavily sampled the *gauche* conformation typical of the open state (~-51°), but in the WT Hxk2 *holo* simulation, it shifted more to the *anti* conformation typical of the closed state (~160°, Fig 7B and 7C) [55]. Interestingly, the same shift was not observed in the Hxk2^G238V^ simulations, suggesting the mutation at position 238 might impede V236 rotation.

#### D211: catalytic residue

Hxk2^G238V^ also impacts the dynamics of D211, a residue that plays a critical role in catalysis (Figs 6 and 7B). In the glucose-bound state, our ΔRMSF calculations suggest that Hxk2^G238V^ has little impact on D211 flexibility. In contrast, in the unbound state, Hxk2^G238V^ enhances D211 flexibility over WT Hxk2 (Tables S1 and A in S1 Text). The Hxk2^G238V^ D211 samples much of the expected “glucose-ready” conformation (Fig 7B, in blue), but it also flips away from the ATP binding pocket as the ɑ5 helix to which it belongs unfolds slightly (Fig 7B, in pink). This added D211 conformational flexibility could also contribute to reduced catalysis.

#### ɑ11’ helix

Hxk2^G238V^ impacts the local dynamics of a critical alpha helix (ɑ11’) that lines the ATP-binding site (Fig 6) [7]. The ΔRMSF analysis suggests that in the unbound state, the Hxk2^G238V^ enhances the flexibility of this helix over WT Hxk2 (e.g., S419, V420, Y421, and R423). In contrast, in the glucose-bound state, the mutation has little impact on the flexibility of these residues (Tables S1 and A in S1 Text).

### *Sc*Hxk2^G238V^ affects global protein dynamics

Having examined the impact of *Sc*Hxk2^G238V^ on the local dynamics of the enzymatic cleft, we next examined its effect on the global domain-closure dynamics associated with glucose binding and catalysis. Our simulations ran long enough to sample both open (apo, unbound) and closed (holo, glucose bound) conformations. For example, included among the many conformations sampled throughout the WT *Sc*Hxk2 and *Sc*Hxk2^G238V^ apo simulations were those that differed from the open 1IG8 *Sc*Hxk2 crystallographic conformation [7] by only 0.78 and 0.75 Å, respectively, per heavy-atom backbone root-mean-square deviation (RMSD; Fig D in S1 Text). Similarly, included among the conformations of the WT *Sc*Hxk2 and *Sc*Hxk2^G238V^ glucose-bound simulations were those that differed from the closed 3O8M *Kl*Hxk1 conformation [16] by only 1.13 and 1.18 Å, respectively (Fig D in S1 Text). Because the simulations capture both open and closed conformations, they can reasonably inform a study of the transition between these two states.

#### Hxk2^G238V^ alters large-scale opening and closing motions

Before glucose binding, Hxk2 exists predominantly in an open state (i.e., the helical large subdomain and the ɑ/β small subdomain are positioned such that the enzymatic cleft is accessible [7, 8]). Once the glucose binds, these two domains adopt a closed conformation by rotating relative to each other, thus collapsing the cleft and enveloping the glucose molecule [7, 11] (Fig 1). Hxk2^G238V^ notably impacts these global motions, which substantially affect the entire protein structure.

**Fig 8:**
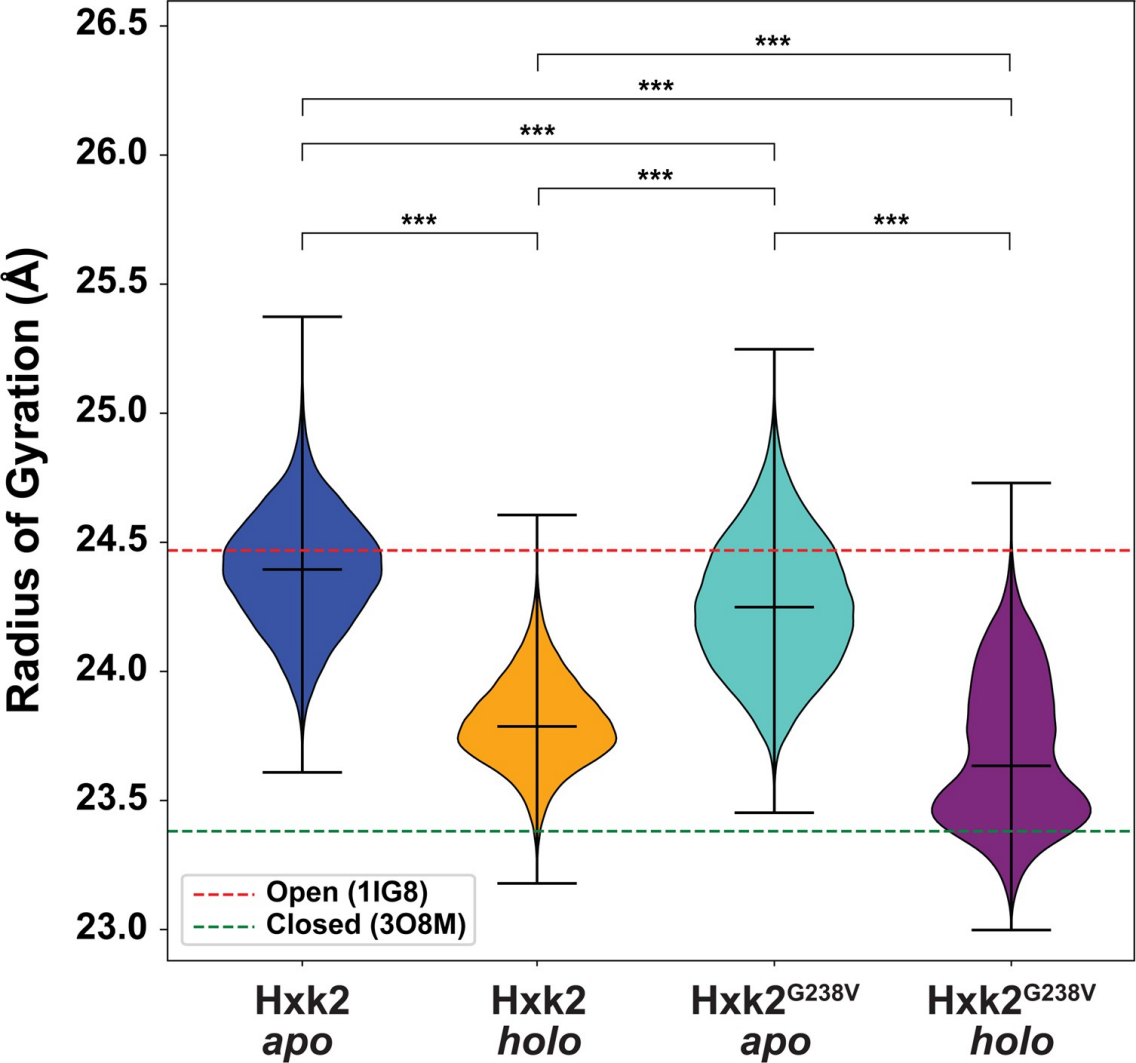
Distributions of the radii of gyration. For reference, the red and green dashed lines show the corresponding values of the 1IG8 (*Sc*Hxk2, open) and 3O8M (*Kl*Hxk1, closed) crystal structures, respectively. The middle horizontal lines correspond to the median values associated with each simulation. The mean of each simulation differs statistically from the means of all others (Kruskal–Wallis, *p*-value < 0.001; Conover post hoc analysis, adjusted *p*-value < 0.001 in all cases, indicated by ***).

To quantify this impact, we calculated the protein radius of gyration (RoG) throughout the four simulations (Fig 8). A larger RoG indicates that Hxk2 is in the open conformation (Fig 8, red dotted line), and a lower RoG indicates the closed conformation (Fig 8, green dotted line). Given that the RoG distributions are not all normally distributed, we performed a non-parametric statistical analysis called the Kruskal-Wallis test [56] to assess differences in means. This analysis led us to reject the hypothesis that there is no difference in the mean RoG values of the Hxk2 *apo*, Hxk2 *holo*, Hxk2^G238V^ *apo*, and Hxk2^G238V^ *holo* simulations (F-statistic: 2.6 x 10^6^; *p*-value < 0.001). A subsequent Conover post hoc analysis [57] revealed that the four simulations are all different in terms of the RoG values sampled (adjusted *p*-value < 0.001 in all cases).

We found that the Hxk2^G238V^ RoG was generally lower than that of WT Hxk2 (Fig 8), suggesting that Hxk2^G238V^ is less prone to adopt a fully open conformation (Cohen’s d values of 0.551 and 0.516 for the apo and holo states, respectively, corresponding to medium effect sizes for both [58]). The standard deviations associated with the Hxk2^G238V^ simulations (both apo and holo) were also greater than those associated with the WT Hxk2 simulations, suggesting that glucose binding to Hxk2^G238V^ is less prone to induce the closed state required for catalysis, perhaps explaining why the Hxk2^G238V^ K_m_ for glucose is ten times higher than that of WT Hxk2 (Table 2).

To further verify the impact of Hxk2^G238V^ on large-scale open-to-closed motions, we used the POVME2 algorithm [59, 60] to measure the volume of the enzymatic cleft over the course of the simulations. The means and standard deviations of the binding-cleft volume showed similar trends (Fig E in S1 Text), corroborating the observed differences in large-scale motions and suggesting that those differences directly impact the shape of the enzymatic cleft.

#### Hxk2^G238V^ alters the centrality of cleft-lining amino acids

Hxk2^G238V^ appears to modify how the impact of glucose binding propagates to distant protein regions (e.g., along pathways of adjacent residues whose motions influence one another), possibly explaining why glucose binding is less likely to induce domain closure. To assess potential shifts in these residue-residue communication pathways, we calculated each residue’s betweenness centrality (BC), which measures the extent to which that residue participates in these many pathways. Said another way, BC indicates how essential a given amino acid is for intra-protein communication (e.g., signal propagation; see the Materials and Methods for a more formal description).

Ensemble-average, per-residue betweenness centrality (BC) calculations [61] revealed that residues of the WT Hxk2 enzymatic cleft have a substantial influence on intra-protein communication. The β9/β10 β-hairpin residues are present in 14.19% and 15.87% of the shortest paths calculated from the WT Hxk2 *apo* and *holo* simulations, respectively, but comprise only 1.28% of the residues in the simulation (I231 to V236, 6/469); D211 is present in 4.96% and 3.70% of the *apo* and *holo* paths, respectively, but comprises only 0.21% of all residues (1/469); and ɑ11’ residues are present in 12.28% and 11.70% of the *apo* and *holo* paths, respectively, but comprise only 1.92% of all residues (D417 to P425, 9/469) (S1 Table).

These same BC calculations applied to Hxk2^G238V^ reveal substantial changes in the influence of cleft-lining residues. The BC of V238 increases, suggesting it has a greater influence on inter-protein communication (large effect size in both the *apo* and *holo* states; Cohen’s *d* values of -2.45 and -2.22, respectively, more than for any other residue). In contrast, the BC of D211 decreases, suggesting reduced influence (medium effect size in *apo* state, Cohen’s *d* value of 0.67, third largest decrease; negligible effect size in the *holo* state, Cohen’s *d* value of 0.11). The impact on the BC of the β9/β10 β-hairpin and ɑ11’ residues is also profound, though more varied. Hxk2^G238V^ increases the BC of some residues while decreasing others (S1 Table).

These results suggest that in WT Hxk2, the enzymatic cleft is a nexus through which much intra-protein communication (“information”) flows. The impact of glucose binding at the cleft can, in theory, readily propagate throughout the protein, inducing domain closure and catalysis. But Hxk2^G238V^ alters the betweenness centrality of the cleft-lining residues, in part by shifting centrality to V238, a residue that does not directly interact with bound glucose. These changes may alter how the glucose-binding signal propagates throughout the protein, impeding domain closure and catalysis.

### Summary of computational results

In summary, though the mutated residue V238 does not directly interact with bound glucose or 2DG (Fig 6), it likely impacts hexose binding and catalysis via allosteric mechanisms. Hxk2^G238V^ increases the flexibility of several key binding-cleft residues (Fig 6), including catalytic residue D211 (Fig 7B) and V236 (Fig 7B and 7C). These changes to local dynamics also influence global motions. Hxk2^G238V^ is less likely to adopt a fully open conformation, and *holo* (glucose-bound) Hxk2^G238V^ is less likely to adopt the closed conformation required for catalysis (Fig 8). Hxk2^G238V^-induced changes in betweenness centrality appear to alter inter-protein communication, perhaps explaining why glucose binding at the enzymatic cleft is less likely to induce domain closure.

## Discussion

In this work, we exposed a drug-sensitive yeast strain to 2DG, a toxic glucose analog. Whole genome sequencing revealed a novel mutation in hexokinase 2 (Hxk2^G238V^) that confers 2DG resistance. We demonstrate that this mutation is sufficient to confer 2DG-resistance and that the *hxk2*^G238V^ allele encodes a stable but hypomorphic protein. This enzyme has reduced catalytic activity toward both glucose and 2DG but appears capable of phosphorylating these molecules *in vivo*, as evidenced by our phenotypic analyses (Figs 2, 3, and 5). The altered residue does not appear to interact directly with glucose or ATP, but Hxk2^G238V^ has higher K_m_ values and reduced specific activity for both substrates. This finding is significant because, to the best of our knowledge, only one other 2DG-resistance *hxk2* mutation has been discovered that does not directly impact the enzymatic cleft (Hxk2^G55V^) [40]. All others alter amino acids that line the glucose-binding (e.g., Hxk2^T212P^, Hxk2^K176T^, and Hxk2^Q299H^ [39]) or ATP-binding (e.g., Hxk2^D417G^, Hxk2^R423T^, Hxk2^D211A^ [40], Hxk2^G418C^, and Hxk2^T75I/S345P^ [39]) pockets.

### Allosteric influences on local and global dynamics

The Hxk2^G238V^ variant substitutes a glycine for a valine, two residues that differ only by an isopropyl group. Yet, the presence of those three additional heavy atoms indirectly influences multiple components of the catalytic mechanism (Fig 7A) via allosteric effects on local dynamics that shift the global conformational ensemble. Indeed, our work provides a dramatic example of how even small changes to protein structure can drastically alter protein function—in this case, catalysis—via allosteric mechanisms. Though V238 does not line the enzymatic cleft, it impinges on the motions of neighboring residues, which in turn impinge on their neighbors, etc., thus (1) propagating a signal to pocket-lining Hxk2^G238V^ residues, (2) perturbing local and global protein dynamics, and (3) ultimately reducing Hxk2^G238V^ catalytic activity.

Our work suggests this allosteric influence alters the flexibility of the enzymatic cleft, possibly increasing the entropic penalty of binding. Hxk2^G238V^ increases the flexibility of the *apo* (ligand-absent) binding cleft (Fig 6A, in blue ribbon; Tables S1 and A in S1 Text), which likely increases the number of sampled microstates. In contrast, Hxk2^G238V^ decreases the flexibility of the *holo* (glucose-bound) cleft (Fig 6B, in red ribbon). The entropic penalty of glucose binding may therefore be greater for Hxk2^G238V^ than for WT Hxk2, though other factors also impact entropy (e.g., the order imposed on ligand and water-molecule movements). Experimental methods such as isothermal titration calorimetry could be used to further assess the impact of the mutation on the entropic and enthalpic contributions to glucose and 2DG binding.

Hxk2^G238V^ may also impede glucose and 2DG phosphorylation by allosterically impacting the dynamics of β-hairpin residue V236. We hypothesize that the domain closure associated with catalysis requires V236 to rotate from a *gauche* conformation (prominent in our more open WT *apo* simulation as well as the open-state 1IG8 [7], 3O80, 3O1W, 3O4W, 4JAX, 3O6W, and 3O5B [16] structures) to an *anti* conformation (prominent in our more closed WT *holo* simulation as well as the closed-state 3O8M structure [16]; Figs 7B, 7C, and 8). But in the Hxk2^G238V^ simulations, V236 was overwhelmingly in the *gauche* conformation typical of the open state, even in the more closed *holo* (glucose-bound) simulation (Figs 7B, 7C, and 8). By limiting V236 rotation, Hxk2^G238V^ makes V236 less likely to adopt the *anti* conformation more typical of the close state. Mutagenesis experiments could further clarify the role of the V236 residue and its flexibility. We hypothesize that if larger amino acids (e.g., isoleucine, tryptophan) were substituted at this position, residue 236 would be less prone to rotate, thus impeding domain closure. In contrast, a glycine substitution at position 236 may have the opposite effect.

Hxk2^G238V^ also alters the dynamics of the catalytic residue D211, perhaps further contributing to the reduced glucose and 2DG phosphorylation we observed. In the Hxk2^G238V^ *apo* (ligand-absent) simulation, D211 is less often in the conformation required to optimally engage a bound glucose. D211 at times even flips away from the ATP binding pocket into a displaced conformation (Fig 7B, in pink) that may directly impede glucose binding via steric hindrance.

These allosteric changes in local dynamics correspond to changes in large-scale dynamics. Our simulations suggest that Hxk2^G238V^ substantially interferes with domain closure (Fig 8), rendering the *holo* protein less able to embrace a bound glucose molecule. Indeed, the two ~250 ns Hxk2^G238V^ *holo* simulations both adopted an open conformation despite the presence of a bound glucose molecule; only the longer ~500 ns Hxk2^G238V^ *holo* simulation remained mostly closed (Fig 8). Changes in intra-protein, residue-residue communication pathways may explain the impaired domain closure. Hxk2^G238V^ shifts the betweenness centrality (i.e., importance for intra-protein communication, see Materials and Methods) [61] of some cleft-lining residues (e.g., D211) to residues that do not interact with the bound glucose (e.g., G238). These changes and others to β-hairpin and ɑ11’ residues may impair propagation of the glucose-binding signal throughout the protein, such that it cannot adequately induce domain closure. Experiments such as double electron-electron resonance (DEER) electron paramagnetic resonance (EPR) spectroscopy applied to doubly spin labeled Hxk2 could verify the impact of Hxk2^G238V^ on the average distance between the small and large domain.

In summary, this work is significant because it reaffirms two crucial insights into allosteric mechanisms. First, many nonfibrous ordered proteins are allosteric (i.e., subject to conformational shifts due to point mutations, ligand binding, or changes in external conditions) [62]. Second, allosteric effects are often mediated via subtle changes in residue-residue pathways of correlated motions, not large-scale conformational changes [63].

### Cancer relevance

**Fig 9:**
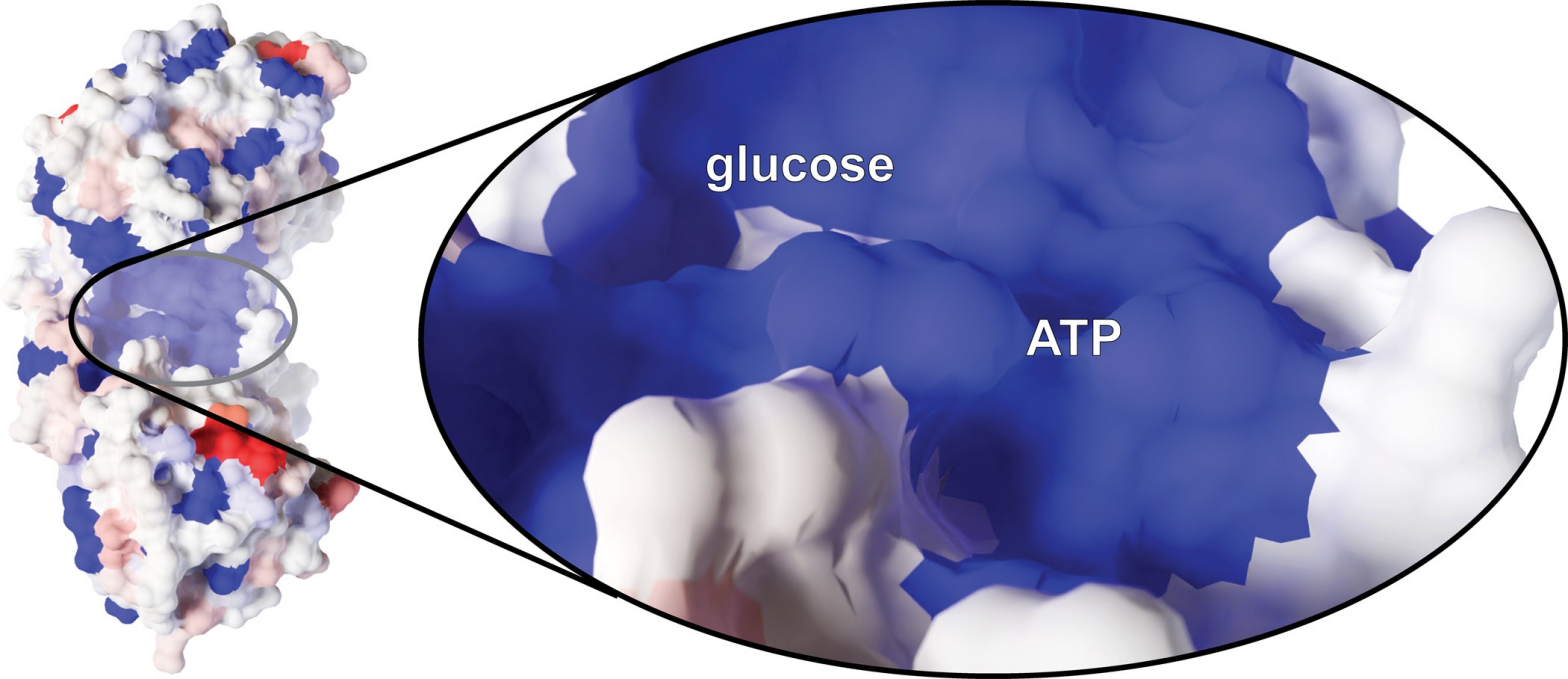
Residue similarity between *Sc*Hxk2 (yeast) and *Hs*Hk2 (human). Similarity is projected onto a structure of *Sc*Hxk2 (PDB 1IG8) and colored per the BLOSUM30 scoring matrix. Highly similar amino acids are shown in blue, and dissimilar amino acids are shown in red.

This work is also significant because human hexokinases are potential cancer drug targets, and *Sc*Hxk2 is an excellent model hexokinase. Hexokinase II (*Hs*Hk2) is arguably the most studied of the four mammalian hexokinase isoforms. *Sc*Hxk2 and *Hs*Hk2 share notable sequence similarity, including ~33% sequence identity per Clustal Omega (Fig C in S1 Text) [53, 54]. The glucose- and ATP-binding sites are even more conserved (Fig 9), suggesting the two proteins have similar mechanisms of action. Phosphorylation of both *Sc*Hxk2 and *Hs*Hk2 increases catalytic activity while simultaneously promoting homodimer dissociation [64].

#### Uncovering 2DG-resistance mechanisms

*Hs*Hk2 is often upregulated in cancer [3, 4, 65, 66]. Some cancer cells rely on glycolysis and lactic acid fermentation for ATP production, even in the presence of adequate oxygen. This tendency towards aerobic glycolysis over the more efficient OXPHOS is known as the Warburg effect [4, 67–71]. *Hs*Hk2 upregulation is critical in such circumstances because it allows cancer cells to drastically increase glycolytic flux so they can maintain ATP levels via glycolysis/fermentation alone [5]. 2DG also binds *Hs*Hk2 and so has anti-cancer properties, both when used alone [22, 72–84] and in combination with other therapies [19, 22, 77–79, 85–107]. Despite this promising therapeutic mechanism, spontaneous resistance to 2DG [74, 85, 108, 109] and other hexokinase inhibitors [74, 108–113] has complicated their use as anti-cancer agents.

To date, extensive genetic screens performed in yeast have revealed the 2DG responsive cellular network [35, 39, 40, 114, 115]. While analogous mapping of the same network in mammalian cells remains to be performed, all the components of the system defined in yeast are conserved in mammals and so can be informative. For example, overexpression of the DOG phosphatases, which dephosphorylate 2DG-6P to counter the activity of Hxk2 in yeast, provide resistance to 2DG in both yeast and HeLa cells [39]. As a second example, we note that the activation of AMPK (the mammalian ortholog of Snf1) after 2DG addition triggers ⍺-arrestin-regulated endocytosis of the Glut1 glucose transporter [115, 116], a pathway that is perfectly conserved in yeast [114, 115]. These facets of the 2DG responsive network surround the activities of hexokinase and in some cases, such as the DOG phosphatases, directly counter its enzymatic function [117].

Given this high degree of pathway conservation, we expect that mutations in mammalian hexokinases (e.g., *Hs*Hk2) will influence 2DG responsiveness just as mutations in yeast Hxk2 do. The existing literature provides some support for this hypothesis. For example, S. Barban developed a 2DG-resistant HeLa cell line that had reduced hexokinase activity for 2DG and glucose, reminiscent of our *Sc*Hxk2^G238V^ variant [118]. Bailey *et al*. also developed a 2DG-resistant pig kidney cell line with an altered rate of 2DG phosphorylation, hinting at the possibility of a 2DG-resistance mutation in hexokinase [119].

Understanding the hexokinase catalytic mechanism and anticipating potential resistance-conferring mutations is critical, given 2DG’s potential as an anti-cancer therapeutic. The present work in *Sc*Hxk2 thus provides insights into the roles that *Hs*Hk2 plays in cancer biology and chemotherapy resistance, insights that will be a driving feature of our future research.

#### Is reduced Hxk2 activity oncogenic in some circumstances?

Given that cancer is generally associated with increased *Hs*Hk2 activity, we were surprised to discover that the COSMIC database [120] reports three *Sc*Hxk2^G238V^-equivalent mutations in HK2 (A236S, A236T, and A684V) that are associated with various carcinomas. These mutations arise independent of 2DG resistance, but given their location it seems likely that they act analogously to *Sc*Hxk2^G238V^ by decreasing *Hs*Hk2 catalytic activity. Hk2 has two catalytically active hexokinase domains [121], so there are two G238V-equivalent positions. Two mutations at the N-terminus position, A236S (COSM4849004) and A236T (COSM3000068), are associated with cervical squamous cell carcinoma and stomach adenocarcinoma, respectively. A mutation at the C-terminus position, A684V (COSM6158814), is associated with lung adenocarcinoma. Although all three Hk2 mutations were also judged pathogenic per the FATHMM algorithm [122], the molecular connection between these mutations and cancer progression remains undefined.

Do these Hk2 mutations promote or impede cancer progression, given that they presumably impair glucose phosphorylation just as *Sc*Hxk2^G238V^ does? There is no data on this point; however, we note that not all cancer cells are subject to the Warburg effect, so elevated Hk2 function may not always be beneficial to cancer cells. For example, although increased Hk2 expression is associated with a more aggressive phenotype in testicular germ cell tumors, overall Hk2 expression is reduced in such tumors relative to paired normal testicular tissues [123]. Per the GEPIA web server [124], the same may be true of lymphoid neoplasm diffuse large B-cell lymphoma, ovarian serous cystadenocarcinoma, acute myeloid leukemia, and thymoma.

While not yet defined molecularly, it is formally possible that reduced Hk2 activity may be advantageous in cancers that have not yet transitioned to a high-glycolytic state. Whether this benefit derives from changes in Glc-6P production or some other Hk2 function (e.g., the protein’s role in apoptosis [125]) is uncertain. Much work remains to better understand the complex and varied roles hexokinases play in normal and disease states.

## Materials and methods

### Yeast strains, plasmids, and growth conditions

Yeast strains employed in this study are listed in Table 3. The strains were grown on YPD (2% peptone, 1% yeast extract, 2% glucose) or synthetic complete medium (per O’Donnell et al. [126]) lacking the amino acids needed for maintaining plasmids. Plasmid information is provided in Table 4. Plasmids were introduced into yeast strains using the lithium acetate transformation method [127]. Where indicated, SC or YPD containing 2% glucose were supplemented with 2DG to a final concentration (presented as % w/v). We generated a 2% 2DG (Sigma-Aldrich, St. Louis MO) stock by dissolving two grams of 2DG in 100 mL of water and then filter sterilizing. Unless otherwise indicated, cells were grown at 30°C.

**Table 3:** Yeast strains used in the current study.

Strain	Genotype	Source
BY4742	MATα his3Δ1 leu2Δ0 lys2Δ0 ura3Δ0	[128]
Parental ABC16-monster (RY0568)	***MAT-α*** *adp1*Δ *snq2*Δ *ycf1Δ pdr15Δ yor1Δ*, *vmr1Δ pdr11Δ*, *nft1Δ bpt1Δ ybt1Δ ynr070wΔ yol075cΔ aus1Δ pdr5Δ pdr10Δ pdr12Δ can1Δ*::*GMToolkit-α* [CMVpr-rtTA NATMX4 STE3pr-LEU2] *his3Δ1 leu2Δ0 ura3Δ0 met15Δ0*	[45]
Naïve ABC16-monster	***MAT-α*** *adp1*Δ *snq2*Δ *ycf1Δ pdr15Δ yor1Δ*, *vmr1Δ pdr11Δ*, *nft1Δ bpt1Δ ybt1Δ ynr070wΔ yol075cΔ aus1Δ pdr5Δ pdr10Δ pdr12Δ can1Δ*::*GMToolkit-α* [CMVpr-rtTA NATMX4 STE3pr-LEU2] *his3Δ1 leu2Δ0 ura3Δ0 met15Δ0*	[45]
2DG Resistant Strain 1 (ABC16-monster)	***MAT-α*** *adp1*Δ *snq2*Δ *ycf1Δ pdr15Δ yor1Δ*, *vmr1Δ pdr11Δ*, *nft1Δ bpt1Δ ybt1Δ ynr070wΔ yol075cΔ aus1Δ pdr5Δ pdr10Δ pdr12Δ can1Δ*::*GMToolkit-α* [CMVpr-rtTA NATMX4 STE3pr-LEU2] *his3Δ1 leu2Δ0 ura3Δ0 met15Δ0*, *hxk2*^*G238V*^	This study.
2DG Resistant Strain 2 (ABC16-monster)	***MAT-α*** *adp1*Δ *snq2*Δ *ycf1Δ pdr15Δ yor1Δ*, *vmr1Δ pdr11Δ*, *nft1Δ bpt1Δ ybt1Δ ynr070wΔ yol075cΔ aus1Δ pdr5Δ pdr10Δ pdr12Δ can1Δ*::*GMToolkit-α* [CMVpr-rtTA NATMX4 STE3pr-LEU2] *his3Δ1 leu2Δ0 ura3Δ0 met15Δ0*, *hxk2*^*G238V*^	This study.
2DG Resistant Strain 3 (ABC16-monster)	***MAT-α*** *adp1*Δ *snq2*Δ *ycf1Δ pdr15Δ yor1Δ*, *vmr1Δ pdr11Δ*, *nft1Δ bpt1Δ ybt1Δ ynr070wΔ yol075cΔ aus1Δ pdr5Δ pdr10Δ pdr12Δ can1Δ*::*GMToolkit-α* [CMVpr-rtTA NATMX4 STE3pr-LEU2] *his3Δ1 leu2Δ0 ura3Δ0 met15Δ0*, *hxk2*^*G238V*^	This study.
2DG Resistant Strain 4 (ABC16-monster)	***MAT-α*** *adp1*Δ *snq2*Δ *ycf1Δ pdr15Δ yor1Δ*, *vmr1Δ pdr11Δ*, *nft1Δ bpt1Δ ybt1Δ ynr070wΔ yol075cΔ aus1Δ pdr5Δ pdr10Δ pdr12Δ can1Δ*::*GMToolkit-α* [CMVpr-rtTA NATMX4 STE3pr-LEU2] *his3Δ1 leu2Δ0 ura3Δ0 met15Δ0*, *hxk2*^*G238V*^	This study.
2DG Resistant Strain 5 (ABC16-monster)	***MAT-α*** *adp1*Δ *snq2*Δ *ycf1Δ pdr15Δ yor1Δ*, *vmr1Δ pdr11Δ*, *nft1Δ bpt1Δ ybt1Δ ynr070wΔ yol075cΔ aus1Δ pdr5Δ pdr10Δ pdr12Δ can1Δ*::*GMToolkit-α* [CMVpr-rtTA NATMX4 STE3pr-LEU2] *his3Δ1 leu2Δ0 ura3Δ0 met15Δ0*, *hxk2*^*G238V*^	This study.
MSY1254	***MAT-α*** *ura3Δ0 leu2Δ0 his3Δ1 hxk2Δ*::*KANMX4*	[40]
MSY1475	***MAT-α*** *ura3Δ0 leu2Δ0 his3Δ1 met15Δ0 hxk1Δ*::*KANMX4 hxk2Δ*::*KANMX4 glk1Δ*::*KANMX4*	[40]

**Table 4:** Plasmid DNA used in the current study.

Name	Description	Source
pRS313	CEN HIS3	[129]
pHxk2-3V5-313	Genomic clone of HXK2 with 592 bp upstream of ATG and 373 bp downstream of the stop and a C-terminal fusion to 3V5; CEN HIS3	This study.
pRS315	CEN LEU2	[129]
pRS315-Hxk2-GFP	Genomic clone of HXK2 with 592 bp upstream of ATG and 373 bp downstream of the stop and a C-terminal fusion to GFP; CEN HIS3	This study.
pRS315-Hxk2-G238V-GFP	Genomic clone of HXK2 with 592 bp upstream of ATG and 373 bp downstream of the stop and a C-terminal fusion to GFP; the G238V mutation was introduced by site-directed mutagenesis; CEN HIS3	This study.
pRS315-Hxk2-3V5	Genomic clone of HXK2 with 592 bp upstream of ATG and 373 bp downstream of the stop and a C-terminal fusion to 3V5; CEN HIS3	[40]
pRS315-Hxk2-G55V-3V5	Genomic clone of HXK2 with 592 bp upstream of ATG and 373 bp downstream of the stop and a C-terminal fusion to 3V5; the G55V mutation was introduced by site-directed mutagenesis; CEN HIS3	[40]
pRS315-Hxk2-G238V-3V5	Genomic clone of HXK2 with 592 bp upstream of ATG and 373 bp downstream of the stop and a C-terminal fusion to 3V5; the G238V mutation was introduced by site-directed mutagenesis; CEN HIS3	This study.

### In vitro evolution and whole genome sequencing analysis

We used directed evolution to identify mutations that confer 2DG resistance to the ABC16-monster strain [38, 42–45], which lacks sixteen ABC transporters. We evolved resistance via serial passaging in five independent replicates. In each passage, cultures were grown at 30°C in 30 mL of YPD (2% peptone, 1% yeast extract, 2% glucose) and 2DG, with shaking at 250 rpm. We stopped each passage when the growth reached saturation (OD ~3.0 per visual inspection) and examined the cultures under a microscope to verify that there was no contamination. We then placed 300 μL aliquots into a fresh supply of 30 mL YPD with 2DG (i.e., a 1:100 dilution into fresh media with drug) and repeated the process. In early passages, 0.05% 2DG was added, and growth to saturation required 4–5 days. As resistance developed, the time needed for saturation shortened to roughly two days. To ensure evolved resistance at higher 2DG concentrations, we then increased the 2DG concentration to 0.2% and resumed serial passages. Each replicate required between eight and twelve passages total, at which point the growth rate of each had stabilized (per eighteen-hour growth curves calculated at multiple 2DG concentrations, 0.05–0.2%). To enable comparative genomics and growth-rate analyses, we also generated a no-drug control that involved passaging for the same time intervals but in medium containing no 2DG.

To determine the genomic changes associated with evolved 2DG resistance, we isolated the genomic DNA of both the resistant (passaged) and control strains using a glass-bead/phenol-extraction protocol [130]. We performed next generation sequencing on Illumina NextSeq500 machines. As detailed in Soncini *et al*. [40], sequencing libraries were prepared and multiplexed into single lanes for each strain to produce 151-bp paired end reads.

To identify potential resistance-conferring mutations, we followed the protocol described in Ellison et al. [131]. In brief, we used the Bowtie 2 software [132] to align the sequence reads to the S288C reference yeast genome. We then used Samtools 1.3.1 [133] to sort the alignments by their leftmost coordinates and to index the sorted alignments. BCFtools 1.3.1 [133] was used for variant calling (consensus calling model). VCFtools 0.1.14 [134] was used to identify variants that differed between the 2DG-resistant and no-drug control strains. Finally, we used SnpEff 4.3p [135] to annotate the identified variants (e.g., frameshift variants, missense variants, stop-gained variants, disruptive inframe insertions, putative impact high/moderate/low, etc.).

### 2-deoxyglucose resistance assays

We monitored resistance to 2DG in three different ways. First, to verify the 2DG resistance of the passaged strains, we performed serial dilution growth assays by plating serial dilutions of yeast cells onto solid agar medium containing the indicated concentrations of 2DG and allowing cells to grow for the time indicated for each figure at 30°C. We compared the growth of the evolved yeast cells to the unpassaged, parental strain (ABC16-monster) or the parental strain that was passaged using medium lacking 2DG (naïve ABC16-monster). Serial dilution growth assays were performed as described in O’Donnell et al. [126]. In brief, we grew cells to saturation overnight in YPD or SC medium, measured the optical density of each culture, and initiated our dilution series with a cell density of A_600_ = 1.0 (or ~1 x 10^7^ cells/mL). We then made five-fold serial dilutions of cells and pinned them onto solid YPD or SC with or without 2DG (0.05%, 0.2%, and 0.4%).

Second, we assessed our 2DG-resistant or control strains (parental ABC16-monster or naïve ABC16-monster, as indicated above) using growth curve analyses [136]. In brief, we grew cells to saturation in YPD or SC medium, washed cells into fresh medium, and inoculated in triplicate into flat-bottom 96-well plates at an A_600_ of 0.05 in the medium indicated (i.e., YPD or SC containing varying concentrations of 2DG). Prepared plates were incubated with shaking in a BioTek Cytation 5 plate reader (BioTek instruments; Winooski, VT, USA), and optical density measurements were taken every 30 minutes for 24 hours using the Gen5 software package. Optical densities measured over time are presented with a path-length correction (to report measurements in a 1 cm path length). We used these curves to calculate the doubling times of yeast cells via the following equation:

$$doubling\;time=\frac{\ln\left(2\right)}{\left(\frac{\ln({OD}_{2})-\ln\left({OD}_{1}\right)}{t_{2}-t_{1}}\right)}$$

Doubling times were calculated based on the mean growth curves of each strain by selecting two points that span the linear range of the logarithmic growth portion of the growth curve.

Third, we challenged cells with a range of 2DG concentrations, as described in Soncini et al. [40]. In this approach, overnight cultures are grown to saturation in either glucose (Fig 3A) or galactose (Fig 5D) as a carbon source, diluted to an A_600_ of 0.1, and grown in the absence or presence of 2DG (0.01%, 0.02%, 0.05%, 0.1%, or 0.2%) for 18 hours at 30°C with either 2% glucose (Fig 3A) or 2% galactose (Fig 5D) as a carbon source [40]. Each A_600_ was measured, and cell growth was normalized to growth in the absence of 2DG for each strain. The average of three replicate cultures is presented in Fig 3A, with statistical comparisons made using the Student’s t-test for unpaired variables with equal variance. In this case, *p*-values are indicated as follows: * *p* < 0.05, ** *p*< 0.01, *** *p* < 0.001.

### Immunoblotting to assess Hxk2^G238V^ abundance and stability

To assess Hxk2^G238V^ abundance in cells, we performed whole cell protein extracts using the trichloroacetic acid (TCA) method [126, 137]. In brief, an equal density of mid-log phase cells was harvested by centrifugation, washed in water, and then resuspended in water with 0.25 M sodium hydroxide and 72 mM β-mercaptoethanol. Samples were then incubated on ice, and proteins were precipitated by the addition of TCA. After incubation on ice, proteins were collected as a pellet by centrifugation, the supernatant was removed, and the proteins were solubilized in 50 μL of TCA sample buffer (40 mM Tris-Cl [pH 8.0], 0.1 mM EDTA, 8M urea, 5% SDS, 1% β-mercaptoethanol, and 0.01% bromophenol blue). Samples were then heated to 37°C for 30 minutes, and the insoluble material was removed by centrifugation before resolving samples by SDS-PAGE. Proteins were transferred to a membrane support and detected with either anti-GFP antibodies (Santa Cruz Biotechnology) or an anti-V5 probe (Invitrogen), followed by goat anti-mouse IRDye 680 (Thermo) or goat anti-rabbit IRDye 800 (LiCor). Antibody complexes were visualized using an Odyssey Infrared Imager (LiCor), and bands were quantified using the Odyssey software. REVERT (LiCor) total protein staining of membranes was used as a protein loading and membrane transfer control in immunoblotting.

### Enzymatic assays for Hxk2 function

To verify that the *hxk2*^G238V^ mutation alone is sufficient to confer 2DG resistance, we used site-directed mutagenesis to introduce the *hxk2*^G238V^ mutation into a plasmid encoding the *HXK2* gene (Table 4). We performed DNA sequencing of the entire open reading frame to ensure that no unintentional changes were generated. We separately transformed plasmids expressing WT *HXK2* and *hxk2*^G238V^ into the hxk1Δ hxk2Δ glk1Δ triple deletion cells (Table 4) and measured the hexokinase activity associated with these two alleles, as described in Soncini *et al*. [40]. In summary, we prepared protein extracts using a glass-bead extraction protocol and assayed enzymatic activity by coupling the phosphorylation of glucose to its oxidation by glucose-6-phosphate dehydrogenase. The resulting production of NADPH, detected by measuring absorbance at 340 nm, correlates with hexokinase activity. For comparison, we used the same protocol to assess the enzymatic activity of WT Hxk2 (positive control). To measure the Michaelis-Menten constant (K_m_), we measured the reaction rate (v) at several glucose or 2DG concentrations ([S]) using a constant concentration of ATP (1 mM) and plotted the inverse of rate (1/v) against the inverse of concentration (1/[S]) (Lineweaver-Burk plot) [40]. To calculate the K_m_ for ATP, we measured the reaction rate at several ATP concentrations, kept the glucose concentration constant (2 mM), and plotted the inverse rate against the inverse of the substrate concentration.

### Invertase assays

The invertase activity of cells grown in 2% glucose, where expression of the *SUC2* gene that encodes invertase is repressed in an Hxk2-dependent manner, was measured as in Soncini *et al*. [40]. For this assay, three independent cultures were assessed using a colorimetric assay that measures Suc2 enzymatic function coupled to glucose oxidase [138]. The mean of these replicates is plotted with the standard error indicated by the error bars. Invertase activity is measured in units per OD of cells, where 1 unit is equal to 1 μmole of glucose released per minute. Student’s t-test for unpaired variables with equal variance was used to compare the difference between *hxk2*Δ cells containing plasmids expressing WT *HXK2* vs. an empty vector or the *hxk2* mutant alleles. *P*-values are indicated as follows: * p<0.05, ** p<0.01, *** p<0.001.

### Molecular dynamics simulations

To generate models of apo (ligand-absent) Hxk2, we downloaded a crystal structure of Hxk2 from the Protein Data Bank (PDB 1IG8 [7]). We used PDB2PQR 2.1.1 [139, 140] to add hydrogen atoms per the PROPKA algorithm [141] (pH 7.0) and to optimize the hydrogen-bond network. To approximate an in vivo aqueous environment, we used the Ambertools18 program tleap [142] to (1) add a water box extending 10 Å beyond the protein in all directions, (2) add Na+ counter ions to achieve electrical neutrality, and (3) add additional Na+ and Cl- counter ions to approximate a 150 mM solution. A model of Hxk2^G238V^ was prepared similarly, except the glycine at position 238 was first changed to valine using the Mutation-Wizard tool in PyMOL [143].

We similarly generated models of the holo (glucose-bound) systems. There is no crystal structure of glucose-bound *Sc*Hxk2, but the 2YHX structure [144] captures the binding pose of ortho-toluoylglucosamine, a ligand with a glucose-like substructure. This substructure has the same orientation and position as glucose bound to *Sc*Hxk1 (yeast, PDB 3B8A [8]), *Hs*Hk2 (human, PDB 2NZT [121]), and *Hs*Hk1 (human, PDB 4FPB), suggesting that it correctly mimics the *Sc*Hxk2/glucose pose. Given this consistent binding mode, we modeled glucose bound to *Sc*Hxk2 by superimposing a crystallographic glucose bound to ScHxk1 (PDB 3B8A [8]) onto the *Sc*Hxk2 apo model. To prepare all systems for simulation, we used Ambertools18 to parameterize the protein and counter ions per the Amber ff14SB force field [145], and the water molecules per the TIP3P forcefield [146]. To parameterize the glucose molecule for the holo simulations, we used the GLYCAM_06j-1 force field [142].

To relax the geometries of both the WT Hxk2 and Hxk2^G238V^ systems, we applied four rounds of 5,000 minimization steps using NAMD [147, 148]. Each round included progressively more atoms: (1) hydrogen atoms; (2) hydrogen atoms and water molecules; (3) hydrogen atoms, water molecules, and protein side chains; and (4) all atoms. We next equilibrated each apo system using five serial isothermal-isobaric (NPT, 310 K, 1 atm) MD simulations of 0.25 ns each. The first equilibration simulation had a time step of 1.0 fs, and the remaining had time steps of 2.0 fs. We applied progressively weaker restraining forces to the protein backbone atoms at each equilibration step (1.00, 0.75, 0.50, 0.25, and 0.00 kcal/mol/Å^2^, respectively). The holo (glucose-bound) simulations were similarly equilibrated, except we used a single, unrestrained, one ns simulation with a 1.0 fs timestep.

Following minimization and equilibration, we started three independent isothermal-isobaric (NPT, 310 K, 1 atm) productive MD simulations of the WT Hxk2 apo, Hxk2^G238V^ apo, WT Hxk2 holo, and Hxk2^G238V^ holo systems, respectively (twelve simulations total, time steps of 2.0 fs). In each case, two of the simulations ran for roughly 250 ns, and the third ran for roughly 500 ns (Table 5).

**Table 5:** Simulation durations.

System	Simulation 1	Simulation 2	Simulation 3	Total
Hxk2 *apo*	5 ns + 274.79 ns	5 ns + 335.99 ns	5 ns + 495.15 ns	15 ns + 1105.93 ns
Hxk2^G238V^ *apo*	5 ns + 275.55 ns	5 ns + 254.45 ns	5 ns + 511.76 ns	15 ns + 1041.76 ns
Hxk2 *holo*	5 ns + 245.00 ns	5 ns + 245.00 ns	5 ns + 507.13 ns	15 ns + 997.13 ns
Hxk2^G238V^ *holo*	5 ns + 245.00 ns	5 ns + 245.00 ns	5 ns + 495.00 ns	15 ns + 985.00 ns

In all cases, we removed the initial five ns before subsequent analysis. For the sake of clarity, we list this removed segment first, followed by the larger segment used for analysis.

### Confirming that the simulations had fully equilibrated

For each simulation, we aligned trajectory frames taken every ten ps to the corresponding first frame by their backbone heavy atoms. We then used MDAnalysis (V 1.0.0) [149] to calculate the corresponding backbone-heavy-atom RMSD. Plotting these twelve sets of RMSD values over simulation time (Fig F in S1 Text) suggested that the simulations had not fully equilibrated before beginning the productive runs. We discarded the initial five ns pre-equilibrated portions of each simulation. Unless otherwise noted, all subsequent analyses focused only on the equilibrated (retained) portions of the productive runs, using the same 10-ps stride and backbone alignment.

### Root-mean-square fluctuation (RMSF) analyses

To assess the per-residue dynamics of individual amino-acid residues, we used MDAnalysis [149] to calculate the RMSF values of residue centers of geometry, considering trajectory frames taken every ten ps.

### Radius of gyration

To assess the opening and closing of the small and large subdomains (Fig 1), we used MDAnalysis [149] to calculate the protein RoG throughout the simulations. For comparison’s sake, we similarly calculated the crystallographic radii of gyration of *Sc*Hxk2 in the open conformation (PDB 1IG8 [7]) and the closely related *K*. *lactis* Hxk1 in the closed conformation (PDPB 3O8M [16]).

### Dynamic cross-correlation

We calculated DCC matrices using MD-TASK [150]. Values in a DCC matrix describe to what degree the motions of all pairs of C⍺ are correlated (i.e., 1 indicates perfectly correlated motions, and -1 indicates anti-correlated motions). The matrix is calculated as follows:

$$C_{ij}=\frac{\left\langle \Delta r_{i}\cdot \Delta r_{j}\right\rangle }{\sqrt{\left\langle \Delta r_{i}^{2}\right\rangle }\cdot \sqrt{\left\langle \Delta r_{j}^{2}\right\rangle }}$$

where ⟨⟩ denotes time averages over the whole trajectory (frames taken every ten ps), and Δ*r*_*i*_ is the displacement of atom *i* from its average position.

To identify changes in correlated motions due to Hxk2^G238V^, we calculated the element-wise difference between DCC matrices.

### Betweenness centrality

We used MD-TASK to calculate the amino-acid BC values of simulation frames extracted every ten ps [61]. In brief, we represent protein conformations as graphs composed of nodes and edges. The nodes (*n*_*i*_) are the amino-acid Cβ atoms (or Cɑ in the case of glycine), and the edges (*e*_*i*,*j*_) connect any two nodes (*n*_*i*_ and *n*_*j*_) that are within 6.7 Å of each other. By considering all pairs of nodes, one can construct a complete set of the shortest paths between node pairs. The BC of a given node is simply the number of shortest paths from this set that pass through that node. Applying this same calculation to multiple conformations extracted from an MD simulation yields an ensemble-average BC value for each amino-acid node. We calculated these time-average per-residue BC values for each simulation *i*, ⟨BC_*i*_⟩, to account for protein dynamics. Finally, we calculated ⟨BC_*i*_⟩ differences between systems. To assess the effect size of these differences, we used Cohen’s *d* with an in-house script.

### Figure generation

To generate protein-structure images, we set up molecular representations in VMD [151] and imported them into the computer-graphics program Blender using the BlendMol plugin [152]. Select images also include QR codes that encode ProteinVR URLs [153] to enable visualization in stereoscopic 3D (virtual reality).

## Supporting information

S1 Text Additional experimental and computational analyses. The supporting information describes the DNA sequence read depth across all sixteen yeast chromosomes (Fig A); glucose and ATP binding kinetics (Fig B); a sequence alignment of *Sc*Hxk2, *Kl*Hxk1, and the two domains of the *Hs*Hk2 protein (Fig C); evidence that the simulations collectively sample both open and closed conformations (Fig D); the distributions of the simulated enzymatic-cleft volumes (Fig E); the heavy-atom backbone RMSD values between the first and subsequent frames of each simulation (Fig F); further description of the impact of Hxk2^G238V^ on the dynamics of the β9/β10 β-hairpin, D211 catalytic, and ɑ11’ residues (Table A); and the average RMSF and B-factor values of the Hxk2 simulations (Table B).’
(PDF)

S1 Table Measured differences between the WT Hxk2 and Hxk2^G238V^ simulations. The table includes per-residue differences in RMSF, calculated B-factors, dynamic cross correlations with residue 238, and betweenness centrality measures.
(XLSX)
