# Supplementary material for: Novel mutation in hexokinase 2 confers resistance to 2-deoxyglucose by altering protein dynamics
Source: PLoS Comput Biol. 2022 Mar 2;18(3):e1009929. doi: 10.1371/journal.pcbi.1009929 (PMC8920189; doi:10.1371/journal.pcbi.1009929)
Supplement: S1 Text — The supporting information describes the DNA sequence read depth across all sixteen yeast chromosomes (Fig A); glucose and ATP binding kinetics (Fig B); a sequence alignment of ScHxk2, KlHxk1, and the two domains of the HsHk2 protein (Fig C); evidence that the simulations collectively sample both open and closed conformations (Fig D); the distributions of the simulated enzymatic-cleft volumes (Fig E); the heavy-atom backbone RMSD values between the first and subsequent frames of each simulation (Fig F); further description of the impact of Hxk2G238V on the dynamics of the β9/β10 β-hairpin, D211 catalytic, and ɑ11’ residues (Table A); and the average RMSF and B-factor values of the Hxk2 simulations (Table B).’ (PDF) [file pcbi.1009929.s001.pdf]

Analysis of whole genome sequencing to assess chromosomal abundances

Changes in ploidy, which are generally well tolerated in *S. cerevisiae*, can also produce 2DG resistance. As described in studies currently under review, we have evidence that selective amplifications or deletions of chromosomal regions are tied to 2DG resistance [1]. All five 2DG-resistant strains in the current work have normal ploidy for each chromosome (Fig A in S1 Text), further showing that Hxk2^{G238V} is responsible for 2DG resistance in these strains.

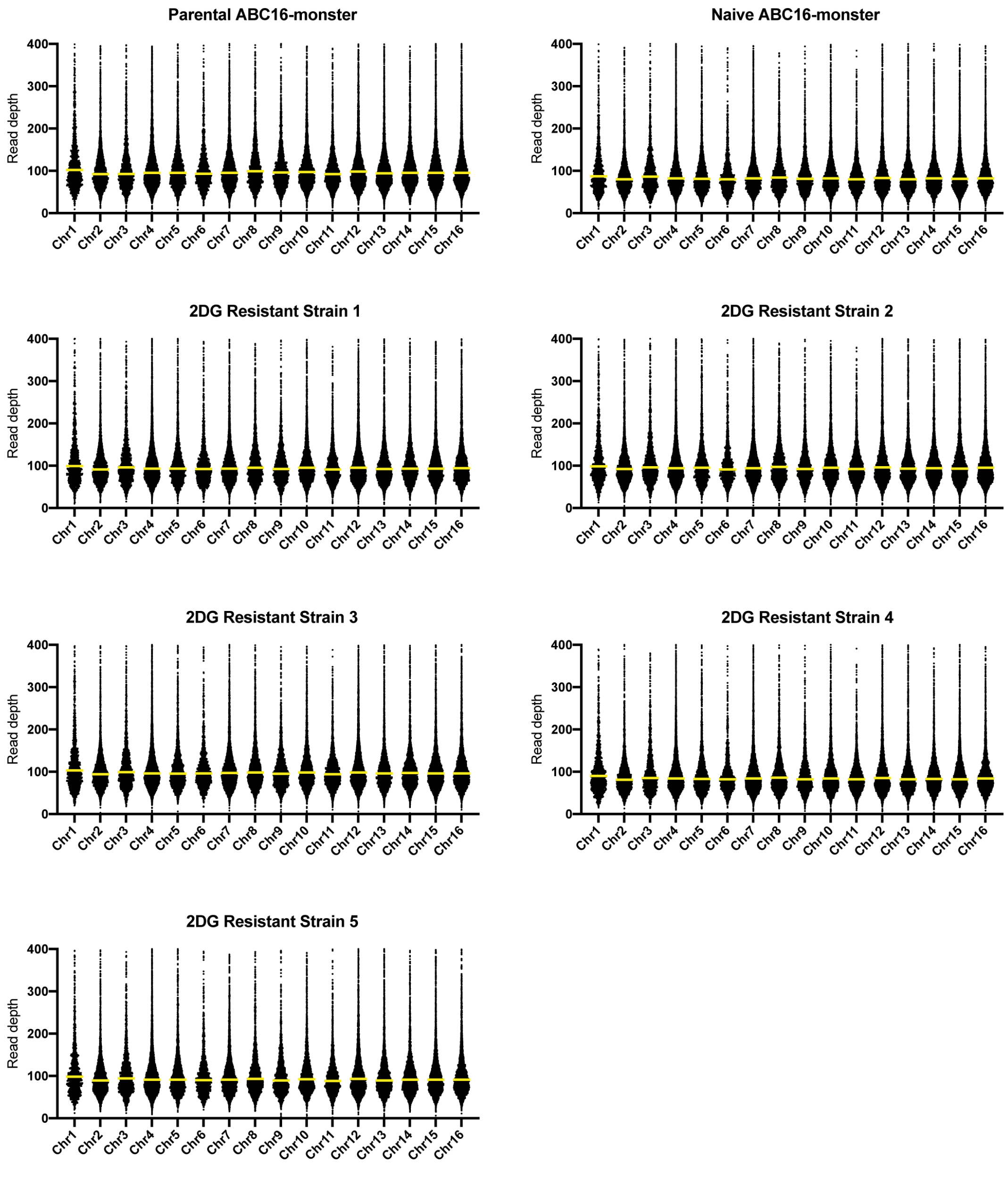

Figure A. DNA sequence read depth across all sixteen chromosomes. Data was plotted for the parental ABC16-monster, naïve ABC16-monster, and 2DG-resistant strains. The median value for each chromosome is shown as a yellow bar.

G238V mutation impacts glucose phosphorylation and ATP hydrolysis

Hxk2^{G238V} adversely impacts both glucose phosphorylation and ATP hydrolysis (Table 2), as measured in enzymatic assays of Hxk2 function (see Materials and Methods). The Michaelis-Menton kinetic plots are provided for three replicate experiments. Below each plot, we show the K_m and V_{max} values, where the V_{max} is determined based on arbitrary units of enzyme added from yeast protein extracts. These data support the values given in Table 2. Note that the Hxk2^{G238V} mutant has consistently higher K_m values for both glucose and ATP than the Hxk2 wild-type protein. While 2DG can be phosphorylated by wild-type Hxk2, it also has a higher K_m and reduced V_{max} compared to glucose. Together these data demonstrate that the *hxk2*^{G238V} allele is hypomorphic, unable to phosphorylate glucose as effectively as Hxk2 and less able to use ATP.

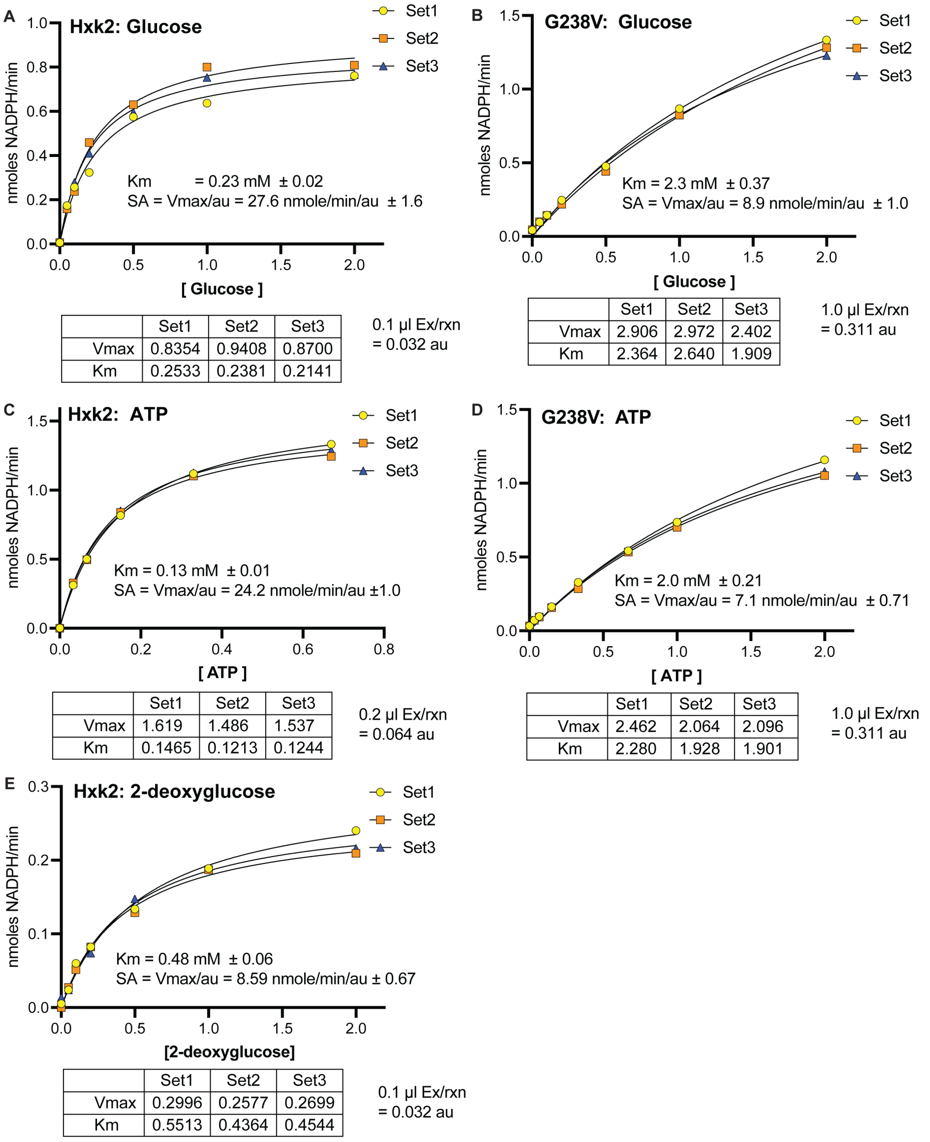

(A)

	Set1	Set2	Set3
Vmax	0.8354	0.9408	0.8700
Km	0.2533	0.2381	0.2141

0.1 µl Ex/rxn = 0.032 au

(B)

	Set1	Set2	Set3
Vmax	2.906	2.972	2.402
Km	2.364	2.640	1.909

1.0 µl Ex/rxn = 0.311 au

(C)

	Set1	Set2	Set3
Vmax	1.619	1.486	1.537
Km	0.1465	0.1213	0.1244

0.2 µl Ex/rxn = 0.064 au

(D)

	Set1	Set2	Set3
Vmax	2.462	2.064	2.096
Km	2.280	1.928	1.901

1.0 µl Ex/rxn = 0.311 au

(E)

	Set1	Set2	Set3
Vmax	0.2996	0.2577	0.2699
Km	0.5513	0.4364	0.4544

0.1 µl Ex/rxn = 0.032 au

Figure B. Glucose and ATP binding kinetics. The data in these graphs support the enzymatic activity reported in Table 2. Three replicate experiments for each assay were preformed, and the results of each individual assay are presented here. In each case, total protein extracts were made from *hxk1*Δ *hxk2*Δ *glk1*Δ cells containing either WT Hxk2 (panels A, C and E) or Hxk2-G238V (panels D and B) and used in enzymatic assays where NADPH production is a readout for Hxk2 enzymatic function, as described in the Materials and Methods. (A) WT Hxk2 enzyme kinetics of glucose turnover were measured using 0.1 µl of yeast protein extract, which corresponds to 0.032 au of enzyme. (B) Hxk2-G238V enzyme kinetics of glucose turnover were measured using 1.0 µl of yeast protein extract, which corresponds to 0.311 au of enzyme. (C) WT Hxk2 enzyme kinetics of ATP turnover were measured using 0.2 µl of yeast protein extract, which corresponds to 0.064 au of enzyme. (D) Hxk2-G238V enzyme kinetics of ATP turnover were measured using 1.0 µl of yeast protein extract, which corresponds to 0.064 au of enzyme. (E) WT Hxk2 kinetics of 2DG turnover were measured using 0.1 µl of yeast protein extract, which corresponds to 0.032 au of enzyme. A similar assay with Hxk2-G238V and 2DG did not yield values above the lower limits of detection for this assay.

Sequence alignments of select hexokinases in yeasts and humans

We used Clustal Omega [2, 3] to align the amino-acid sequences of *Sc*Hxk2, *Kl*Hxk1, and the two domains of *Hs*Hk2 (Fig C in S1 Text).

```
ScHxk2p                 MVHLGPKKPQARKGSMADVPKELMQQIENFEKIFTVPTETLQAVTKHFISELEKGLSKKG  60
KlHxk1p                 MVRLGPKKPPARKGSMADVPANLMEQIHGLETLFTVSSEKMRSIVKHFISELDKGLSKKG  60
HsHk2p_NTerm            --------------------HDQVQKVDQYLYHMRLSDETLLEISKRFRKEMEKGLGATT  40
HsHk2p_CTerm            --------------------ADQHRARQKTLEHLQLSHDQLLEVKRRMKVEMERGLSKET  40
                                             :  .  .     : :  : :  : :::  *:::**.

ScHxk2p                 ---GNIPMIPGWVMDFPTGKESGDFLAIDLGGTNLRVVLVKLGGD--RTFDTTQSKYRLP  115
KlHxk1p                 ---GNIPMIPGWVVEYPTGKETGDFLALDLGGTNLRVVLVKLGGN--HDFDTTQNKYRLP  115
HsHk2p_NTerm            HPTAAVKMLPTFVRSTPDGTEHGEFLALDLGGTNFRVLWVKVTDNGLQKVEMENQIYAIP  100
HsHk2p_CTerm            HASAPVKMLPTYVCATPDGTEKGDFLALDLGGTNFRVLLVRVRNGKWGGVEMHNKIYAIP  100
                           . : *:* :*   * *.* *:***:******:**: *:: ..    .:  :. * :*

ScHxk2p                 DAMRTTQNPDELWEFIADSLKAFIDEQFPQGISEPIPLGFTFSFPASQNKINEGILQRWT  175
KlHxk1p                 DHLRTG-TSEQLWSFIAKCLKEFVDEWYPDGVSEPLPLGFTFSYPASQKKINSGVLQRWT  174
HsHk2p_NTerm            EDIMRG-SGTQLFDHIAECLANFMDKLQIKD--KKLPLGFTFSFPCHQTKLDESFLVSWT  157
HsHk2p_CTerm            QEVMHG-TGDELFDHIVQCIADFLEYMGMKG--VSLPLGFTFSFPCQQNSLDESILLKWT  157
                        : :    .  :*:..*...:  *::    ..    :*******:*. *..::...*  **

ScHxk2p                 KGFDIPNIENHDVVPMLQKQITKR-NIPIEVVALINDTTGTLVASYYTDPETKMGVIFGT  234
KlHxk1p                 KGFDIEGVEGHDVVPMLQEQIEKL-NIPINVVALINDTTGTLVASLYTDPQTKMGIIIGT  233
HsHk2p_NTerm            KGFKSSGVEGRDVVALIRKAIQRRGDFDIDIVAVVNDTVGTMMTCGYDDHNCEIGLIVGT  217
HsHk2p_CTerm            KGFKASGCEGEDVVTLLKEAIHRREEFDLDVVAVVNDTVGTMMTCGFEDPHCEVGLIVGT  217
                        ***.  . *..*** :::: * :  :: :::**::***.**:::. : * . ::*:*.**

ScHxk2p                 GVNGAYYDVCSDIEKLQGKLSDDIPPSAPMAINCEYGSFDNE-HVVLPRTKYDITIDEES  293
KlHxk1p                 GVNGAYYDVVSGIEKLEGLLPEDIGPDSPMAINCEYGSFDNE-HLVLPRTKYDVIIDEES  292
HsHk2p_NTerm            GSNACYMEEMRHIDMVEG-------DEGRMCINMEWGAFGDDGSLNDIRTEFDQEIDMGS  270
HsHk2p_CTerm            GSNACYMEEMRNVELVEG-------EEGRMCVNMEWGAFGDNGCLDDFRTEFDVAVDELS  270
                        * *..* :    :: ::*        .. *.:* *:*:*.::  :   **::*  :*  *

ScHxk2p                 PRPGQQTFEKMSSGYYLGEILRLALMDMYKQGFIFKNQDLSKFDKPFVMDTSYPARIEED  353
KlHxk1p                 PRPGQQAFEKMTSGYYLGEIMRLVLLDLYDSGFIFKDQDISKLKEAYVMDTSYPSKIEDD  352
HsHk2p_NTerm            LNPGKQLFEKMISGMYMGELVRLILVKMAKEELLFGGKLSPELLNTGRFETKDISDIEGE  330
HsHk2p_CTerm            LNPGKQRFEKMISGMYLGEIVRNILIDFTKRGLLFRGRISERLKTRGIFETKFLSQIESD  330
                         .**:* **** ** *:**::*  *:.: .  ::* .:   .:     ::*.  : **  :

ScHxk2p                 PFENLEDTDDLFQNEFGINTTVQERKLIRRLSELIGARAARLSVCGIAAICQKRGYK---  410
KlHxk1p                 PFENLEDTDDLFKTNLNIETTVVERKLIRKLAELVGTRAARLTVCGVSAICDKRGYK---  409
HsHk2p_NTerm            KDGIRKAREV--LMRLGLDPTQEDCVATHRICQIVSTRSASLCAATLAAVLQRIKENKGE  388
HsHk2p_CTerm            CLALLQVRAI--LQHLGLESTCDDSIIVKEVCTVVARRAAQLCGAGMAAVVDRIRENRGL  388
                             :        .:.:: *  :    :.:. ::. *:* *  .  ::*: ::   :

ScHxk2p                 ---TGHIAADGSVYNRYPGFKEKAANALKDIYGWTQTSLDDYPIKIVPAEDGSGAGAAVI  467
KlHxk1p                 ---TAHIAADGSVFNRYPGYKEKAAQALKDIYNWDVEKMEDHPIQLVAAEDGSGVGAAII  466
HsHk2p_NTerm            ERLRSTIGVDGSVYKKHPHFAKRLHKTVRRLV-------PGCDVRFLRSEDGSGKGAAMV  441
HsHk2p_CTerm            DALKVTVGVDGTLYKLHPHFAKVMHETVKDLA-------PKCDVSFLQSEDGSGKGAALI  441
                              :..**:::: :* : :   :::: :            : :: :***** ***::

ScHxk2p                 AALAQKRIAEGKSVGIIGA  486
KlHxk1p                 ACLTQKRLAAGKSVGIKGE  485
HsHk2p_NTerm            TA-----------------  443
HsHk2p_CTerm            TA-----------------  443
                        :.
```

Figure C. An alignment of *Sc*Hxk2, *Kl*Hxk1, and the two domains of *Hs*Hk2 reveals their high sequence similarity. The position of residue 238 is marked with a box.

Impact of Hxk2^{G238V} on the dynamics of the β9/β10 β-hairpin, D211 catalytic, and α11' residues

Hxk2^{G238V} impacts the local dynamics of the β9/β10 β-hairpin (I231-V236), the catalytic residue D211, and the α11' helix (D417-P425). It also impacts the extent to which β-hairpin and residue-238 motions are correlated (per DCC).

Table A. The impact of Hxk2^{G238V} on the dynamics of the β9/β10 β-hairpin, D211 catalytic, and α11' residues.

Location	Residue	*apo* ΔRMSF	*holo* ΔRMSF	*apo* ΔDCC	*holo* ΔDCC
β9/β10 β-hairpin	I231	-0.13 (0.68-0.82)	0.00 (0.57-0.57)	-0.04 (0.65-0.69)	-0.04 (0.64-0.68)
	F232	-0.33 (0.73-1.06)	-0.07 (0.65-0.72)	-0.05 (0.50-0.55)	0.07 (0.58-0.51)
	G233	-0.25 (1.26-1.50)	0.00 (0.87-0.87)	-0.03 (0.28-0.31)	-0.11 (0.21-0.32)
	T234	-0.59 (1.06-1.66)	**0.49 (1.44-0.95)**	-0.14 (0.18-0.32)	**-0.30 (0.04-0.33)**
	G235	-0.22 (1.04-1.26)	**0.86 (1.81-0.95)**	**-0.28 (0.10-0.38)**	**-0.28 (0.08-0.36)**
	V236	0.12 (0.92-0.80)	0.41 (1.19-0.78)	**-0.41 (0.21-0.62)**	-0.21 (0.35-0.56)
D211 (catalytic)	D211	-0.66 (0.67-1.33)	0.00 (0.57-0.57)	-0.02 (0.23-0.25)	-0.20 (0.34-0.54)
α11' helix	D417	-0.09 (0.67-0.77)	0.00 (0.61-0.61)	-0.08 (0.47-0.55)	-0.07 (0.47-0.54)
	G418	-0.14 (0.82-0.96)	0.05 (0.71-0.66)	0.03 (0.41-0.37)	-0.03 (0.41-0.44)
	S419	-0.55 (1.03-1.58)	0.11 (0.92-0.81)	0.05 (0.29-0.24)	-0.08 (0.21-0.29)
	V420	-0.64 (0.89-1.53)	0.03 (0.83-0.80)	0.04 (0.27-0.24)	-0.09 (0.28-0.37)
	Y421	-0.67 (0.80-1.47)	-0.01 (0.81-0.82)	0.13 (0.29-0.16)	-0.10 (0.30-0.40)
	N422	-0.39 (0.96-1.35)	0.06 (0.98-0.92)	0.17 (0.25-0.07)	-0.09 (0.23-0.32)
	R423	**-0.97 (1.03-1.99)**	0.05 (0.98-0.93)	0.10 (0.19-0.10)	-0.12 (0.18-0.29)
	Y424	-0.24 (0.87-1.11)	-0.17 (1.04-1.22)	-0.04 (0.17-0.21)	-0.19 (0.17-0.36)
	P425	-0.05 (1.00-1.05)	-0.47 (1.60-2.06)	-0.06 (0.04-0.10)	-0.23 (0.12-0.35)

Location	Residue	*apo* ΔB-factor	*holo* ΔB-factor
β9/β10 β-hairpin	I231	-5.53 (12.17 - 17.70)	0.00 (8.55 - 8.55)
	F232	-15.54 (14.03 - 29.57)	-2.52 (11.12 - 13.64)
	G233	-17.44 (41.78 - 59.22)	0.00 (19.92 - 19.92)
	T234	-42.95 (29.57 - 72.52)	**30.82 (54.57 - 23.75)**
	G235	-13.31 (28.47 - 41.78)	**62.47 (86.22 - 23.75)**
	V236	5.44 (22.28 - 16.84)	21.26 (37.27 - 16.01)
D211 (catalytic)	D211	-34.75 (11.81 - 46.56)	0.00 (8.55 - 8.55)
α11' helix	D417	-3.79 (11.81 - 15.60)	0.00 (9.79 - 9.79)
	G418	-6.56 (17.70 - 24.26)	1.81 (13.27 - 11.46)
	S419	-37.78 (27.92 - 65.7)	5.01 (22.28 - 17.27)
	V420	-40.76 (20.85 - 61.61)	1.29 (18.13 - 16.84)
	Y421	-40.03 (16.84 - 56.87)	-0.43 (17.27 - 17.70)
	N422	-23.71 (24.26 - 47.97)	3.00 (25.28 - 22.28)
	R423	**-76.31 (27.92 - 104.23)**	2.52 (25.28 - 22.76)
	Y424	-12.51 (19.92 - 32.43)	-10.70 (28.47 - 39.17)
	P425	-2.70 (26.32 - 29.02)	-44.31 (67.38 - 111.69)

The differences in RMSF and DCC values (ΔRMSF and ΔDCC, respectively), followed by the corresponding raw WT and Hxk2^{G238V} values in parentheses. Negative and positive ΔRMSF values suggest Hxk2^{G238V} has increased or decreased the flexibility of the corresponding residue, respectively. Similarly, negative and positive ΔDCC values suggest that Hxk2^{G238V} has increased or decreased the degree of correlation between the motions of G/V238 and the corresponding residue, respectively. For convenience, we also show the differences in calculated B-factors, derived from the RMSF values using the equation $B_i = (8\pi^2/3)RMSF_i^2$ (see reference [4]). In all cases, values that deviate from the respective means by more than two standard deviations are shown in bold. All values are rounded to the nearest hundredth. See also S1 Table.

Large-scale domain-closure dynamics

Our simulations ran long enough to sample both open (*apo*, unbound) and closed (*holo*, glucose bound) conformations (Fig D in S1 Text).

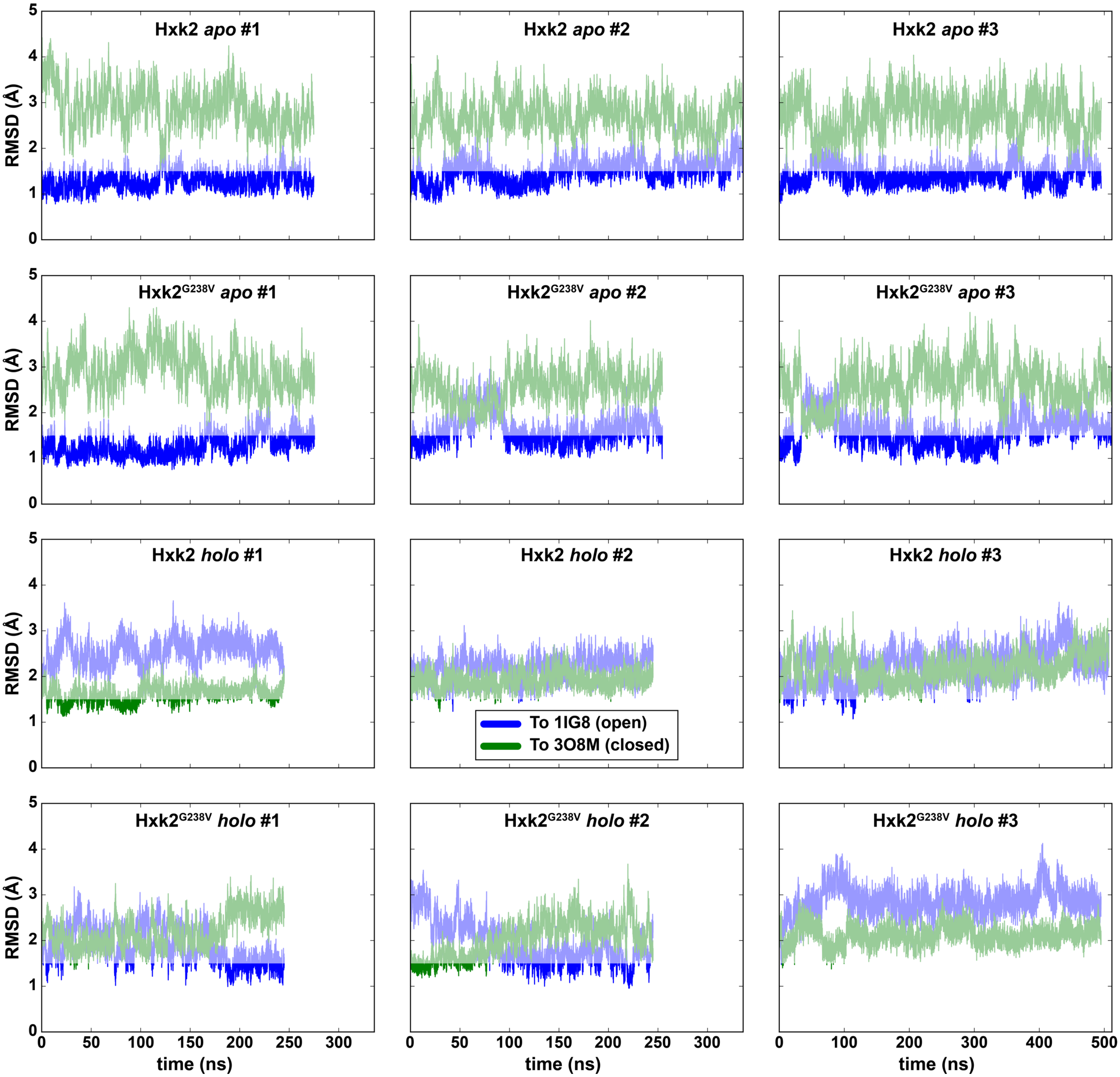

Figure D. The simulations collectively sample both open and closed conformations. The RMS distance between simulated conformations and the open (*Sc*Hxk2, PDB ID: 1IG8 [5]) and closed (*Kl*HXK1, PDB ID: 3O8M [6]) conformation, are shown in blue and green, respectively. The three simulations associated with the Hxk2 *apo*, Hxk2^{G238V} *apo*, Hxk2 *holo*, and Hxk2^{G238V} *holo* systems are shown on the first, second, third, and fourth row, respectively. RMSD values less than 1.5 Å are shown in bold to highlight simulated conformations that are notably close to the open (bolded blue) or closed (bolded green) states.

Volumes of the enzymatic pocket

To assess the impact of Hxk2^{G238V} on the shape and volume of the enzymatic cleft, we used the POVME2 algorithm [7, 8] to analyze the cleft geometries of trajectory frames spaced 100 ps apart. We used a POVME grid spacing of 1.0 Å, with inclusion and contiguous-pocket-seed spheres both centered on the bound glucose molecule (radii of 14.0 Å and 2.0 Å, respectively). To speed the calculations, we did not discard points that fell outside the protein-encompassing convex hull, nor did we consider hydrogen atoms. All other POVME parameters were identical to those given in the example file included in the POVME2 download.

We found that the Hxk2^{G238V} cleft volume was generally lower than that of WT Hxk2, both in the *apo* and *holo* (glucose-bound) states (p-value < 0.001 in both cases per a two sample t-test; Fig E in S1 Text). In both cases, the standard deviations associated with the Hxk2^{G238V} simulations (both *apo* and *holo*) were greater than the corresponding standard deviations associated with the WT Hxk2 simulations.

Ensuring the simulation had equilibrated

We aligned trajectory frames taken every ten ps to the corresponding first frame and calculated the backbone-heavy-atom RMSD. Plotting RMSD values over simulation time suggested that the simulations had not fully equilibrated during the beginning portions of the production runs (Fig F in S1 Text). We discarded the initial five ns pre-equilibrated portions of each simulation. Subsequent analyses focused on the remaining portions.

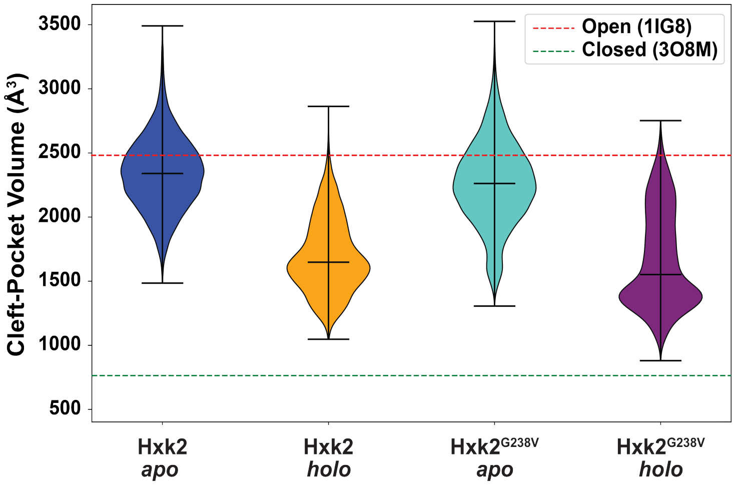

Figure E. Distributions of the enzymatic-cleft volumes. For reference, the red and green dashed lines show the corresponding values of the 1IG8 (*Sc*Hxk2, open) and 3O8M (KlHxk1, closed) crystal structures, respectively. The middle horizontal lines correspond to the median values associated with each simulation.

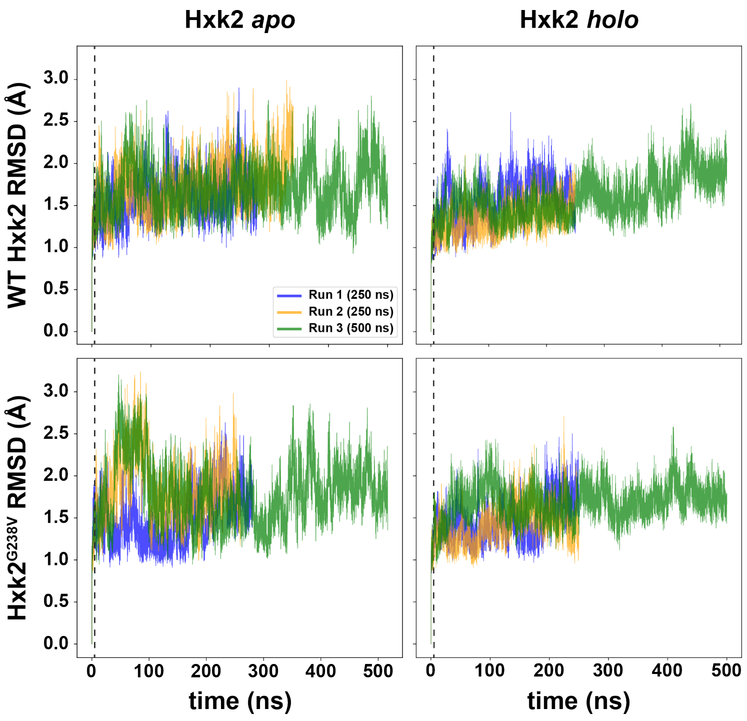

Figure F. The heavy-atom backbone RMSD values between the first and subsequent frames of each simulation. The simulations appear to have converged after five ns (dotted vertical line). Table B in S1 Text similarly provides average RMSF/B-factor values across the 12 simulations.

Average RMSF/B-factor values

We calculated the average RMSF/B-factor values for each of the twelve simulations (4 systems ✕ 3 simulations/system). See Table A in S1 Text for the RMSF/B-factor values of substrate-interacting protein regions, which are more useful for judging impact on ligand binding.

Table B. Average RMSF and B-factor values.

	**Hxk2 *apo***	**Hxk2^{G238V} *apo***	**Hxk2 *holo***	**Hxk2^{G238V} *holo***
**Aggregate**	0.96 ± 0.35<br>(27.44 ± 21.25)	1.12 ± 0.46<br>(38.67 ± 33.41)	1.01 ± 0.39<br>(30.91 ± 24.35)	1.10 ± 0.46<br>(37.42 ± 34.34)
**Run 1**	0.98 ± 0.40<br>(29.79 ± 26.71)	1.00 ± 0.38<br>(30.25 ± 23.96)	0.92 ± 0.39<br>(26.56 ± 25.60)	1.01 ± 0.39<br>(30.60 ± 25.40)
**Run 2**	1.04 ± 0.40<br>(32.85 ± 26.85)	1.02 ± 0.40<br>(31.71 ± 27.48)	0.91 ± 0.37<br>(25.13 ± 22.51)	1.03 ± 0.43<br>(32.46 ± 29.53)
**Run 3**	1.01 ± 0.41<br>(31.07 ± 27.15)	1.12 ± 0.47<br>(38.99 ± 37.75)	1.06 ± 0.42<br>(34.32 ± 27.86)	0.97 ± 0.41<br>(29.05 ± 26.95)

To calculate “Aggregate” values, we concatenated the three simulations associated with each system before calculating RMSF/B-factor values. To calculate “Run 1,” “Run 2,” and “Run 3” values, we considered each of the three simulations separately. The average RMSF value is given first, plus or minus the standard deviation. The average B-factor plus or minus the standard deviation follows in parentheses. B-factors were calculated from the RMSF values using the equation $B_i = (8\pi^2/3)RMSF_i^2$ (see reference [4]). The C- and N-termini were particularly flexible, so we excluded the first five and last five residues of the simulated construct from the calculation (i.e., we included only residues L23 to V481).

References

1. Barney JB, Chandrashekarappa DG, Soncini SR, Schmidt MC. Drug resistance in diploid yeast is acquired through dominant alleles, haploinsufficiency, gene duplication and aneuploidy. PLoS Genetics. 2021;17(9): e1009800.
2. Sievers F, Wilm A, Dineen D, Gibson TJ, Karplus K, Li W, et al. Fast, scalable generation of high-quality protein multiple sequence alignments using clustal omega. Mol Syst Biol. 2011;7: 539.
3. Goujon M, McWilliam H, Li W, Valentin F, Squizzato S, Paern J, et al. A new bioinformatics analysis tools framework at embl-ebi. Nucleic Acids Res. 2010;38(Web Server issue): W695-699.
4. Dauber-Osguthorpe P, Osguthorpe DJ, Stern PS, Moult J. Low frequency motion in proteins: Comparison of normal mode and molecular dynamics of streptomyces griseus protease a. Journal of computational physics. 1999;151(1): 169-189.
5. Kuser PR, Krauchenco S, Antunes OA, Polikarpov I. The high resolution crystal structure of yeast hexokinase pii with the correct primary sequence provides new insights into its mechanism of action. Journal of Biological Chemistry. 2000;275(27): 20814-20821.
6. Kuettner EB, Kettner K, Keim A, Svergun DI, Volke D, Singer D, et al. Crystal structure of hexokinase klhxk1 of kluyveromyces lactis: A molecular basis for understanding the control of yeast hexokinase functions via covalent modification and oligomerization. J Biol Chem. 2010;285(52): 41019-41033.
7. Durrant JD, de Oliveira CAF, McCammon JA. POVME: An algorithm for measuring binding-pocket volumes. Journal of Molecular Graphics and Modelling. 2011;29(5): 773-776.
8. Durrant JD, Votapka L, Sørensen J, Amaro RE. POVME 2.0: An enhanced tool for determining pocket shape and volume characteristics. Journal of chemical theory and computation. 2014;10(11): 5047-5056.
